# Soil exchange rates of COS and CO^18^O differ with the diversity of microbial communities and their carbonic anhydrase enzymes

**DOI:** 10.1038/s41396-018-0270-2

**Published:** 2018-09-13

**Authors:** Laura K. Meredith, Jérôme Ogée, Kristin Boye, Esther Singer, Lisa Wingate, Christian von Sperber, Aditi Sengupta, Mary Whelan, Erin Pang, Marco Keiluweit, Nicolas Brüggemann, Joe A. Berry, Paula V. Welander

**Affiliations:** 1 Department of Earth System Science, Stanford University, Stanford, CA 94305 USA; 2 School of Natural Resources and the Environment, University of Arizona, Tucson, AZ 85721 USA; 3 INRA/Bordeaux Science Agro, UMR 1391 ISPA, Bordeaux Science Agro, Villenave d’Ornon, Bordeaux, 33140 France; 4 SLAC National Laboratory, Stanford Synchrotron Radiation Lightsource, Menlo Park, CA 94025 USA; 5 Department of Energy Joint Genome Institute, Walnut Creek, CA 94598 USA; 6 Institute for Crop Science and Resource Conservation (INRES), Soil Science and Soil Ecology, University of Bonn, Bonn, 53115 Germany; 7 Department of Geography, McGill University, 805 Sherbrooke St. W., Montreal, QC H3A 0B9 Canada; 8 University of Arizona, Biosphere 2, Tucson, AZ 85721 USA; 9 Department of Global Change Ecology, Carnegie Institution for Science, Stanford, CA 94305 USA; 10 Stockbridge School of Agriculture, University of Massachusetts, Amherst, MA 01003 USA; 11 Forschungszentrum Jülich, Institute of Bio- and Geosciences, Agrosphere (IBG-3), Wilhelm-Johnen-Strasse, Jülich, 52428 Germany

**Keywords:** Biogeochemistry, Metagenomics, Microbial ecology

## Abstract

Differentiating the contributions of photosynthesis and respiration to the global carbon cycle is critical for improving predictive climate models. Carbonic anhydrase (CA) activity in leaves is responsible for the largest biosphere-atmosphere trace gas fluxes of carbonyl sulfide (COS) and the oxygen-18 isotopologue of carbon dioxide (CO^18^O) that both reflect gross photosynthetic rates. However, CA activity also occurs in soils and will be a source of uncertainty in the use of COS and CO^18^O as carbon cycle tracers until process-based constraints are improved. In this study, we measured COS and CO^18^O exchange rates and estimated the corresponding CA activity in soils from a range of biomes and land use types. Soil CA activity was not uniform for COS and CO_2_, and patterns of divergence were related to microbial community composition and CA gene expression patterns. In some cases, the same microbial taxa and CA classes catalyzed both COS and CO_2_ reactions in soil, but in other cases the specificity towards the two substrates differed markedly. CA activity for COS was related to fungal taxa and β-D-CA expression, whereas CA activity for CO_2_ was related to algal and bacterial taxa and α-CA expression. This study integrates gas exchange measurements, enzyme activity models, and characterization of soil taxonomic and genetic diversity to build connections between CA activity and the soil microbiome. Importantly, our results identify kinetic parameters to represent soil CA activity during application of COS and CO^18^O as carbon cycle tracers.

## Introduction

COS and CO^18^O are atmospheric tracers for partitioning the net exchange of CO_2_ over land into its respiratory and photosynthetic components, which is critical for benchmarking predictions of climate-carbon feedbacks and their impacts on large terrestrial carbon stocks [1, 2]. The basis of these tracers is their reaction with carbonic anhydrase enzymes (CAs) in leaves, which facilitate photosynthetic carbon fixation by catalyzing the reversible hydration of carbon dioxide (CO_2_+H_2_O⇔HCO_3_^−^+H^+^). CAs are not limited to plants [3], and are present in soil-dwelling microorganisms [4]. In leaves and soils, CAs drive COS consumption and CO_2_–H_2_O isotopic equilibration, and this activity influences patterns in atmospheric composition that can be used to constrain terrestrial photosynthesis. Specifically, during CO_2_ hydration, oxygen isotopes are exchanged between CO_2_ and water molecules in leaves and soils (CO_2_+H_2_^18^O⇔CO^18^O+H_2_O) [5–7], thereby influencing the concentration of CO^18^O in the atmosphere [8, 9]. Consequently, variations of atmospheric CO^18^O reflect the extent of CO_2_ interaction with the leaf and soil water pools, and CO^18^O can be used to trace land photosynthesis and soil respiration at large scales [10–13]. CAs also catalyze the irreversible hydrolysis of COS in leaves and soils (COS+H_2_O→CO_2_+H_2_S). As COS uptake by the terrestrial biosphere is dominated by photosynthetic uptake during the growing season [14], fluctuations of atmospheric COS concentration can also serve as a tracer of land photosynthesis, independently of those from CO^18^O [14–22].

Soil exchange rates of COS and CO^18^O are significant and the drivers of their variability are not well understood. Biological activity in soils has been observed to accelerate oxygen isotope exchange between atmospheric CO_2_ and soil water 10 to 1000 times above uncatalyzed CO_2_ hydration rates [13, 23–26] and to drive significant COS uptake in a variety of ecosystems [27–29]. Soil exchange of COS and CO^18^O [9, 13, 23, 24, 28, 30–34] and soil CA activity [13, 35] are spatially and temporally variable, which introduces uncertainty during the inversion of atmospheric CO^18^O and COS to estimate primary productivity. Systematic investigations of soil CA activity for COS and CO^18^O are needed to determine how ecological and environmental drivers impact soil exchange rates.

Knowledge of the key microbial taxa and CA diversity that drive soil CA activity for COS and CO_2_ is needed to improve their mechanistic understanding and model representation. CAs are diverse and widespread enzymes that include six known classes (α, β, γ, δ, ζ, η) [4, 36]. Organisms often contain CA genes from more than one class or multiple genes encoding CA from the same class. CA are found in autotrophic and heterotrophic microorganisms, and participate in C fixation, pH regulation, and sulfur metabolism [4, 37]. CA activity for CO_2_ has been demonstrated in archaea, bacteria, fungi, algae, plants, and animals [4, 13, 35, 36, 38–40], and COS consumption has been described in bacteria and fungi [37, 41–43]. Recent work found that algal abundance was correlated with soil CO_2_–H_2_O isotope exchange rates and fungal abundance was correlated with soil COS consumption rates [44]—consistent with reductions in COS consumption in soils exposed to fungicides [45]. Thus, while CA are apparently widespread in the soil microbiome, activity for COS and CO_2_ may be primarily driven by specific microbial taxa or CA classes.

Here we report COS and CO^18^O exchange rates in soils collected from a variety of biomes and land uses encompassing a range of soil and environmental properties that we anticipated would influence the soil microbiome. Our approach was to measure gas exchange rates in lab incubations isolated from environmental fluctuations under controlled conditions to characterize the soil and microbial properties driving CA activity. Our null hypothesis was that soil CA activity would vary with soil microbial community structure because of associated differences in the relative abundance and expression profiles of CA classes. We analyzed soil chemical and physical properties, microbial community composition, and CA gene expression profiles and generated robust relationships with soil CA activity derived from CO_2_ and COS trace gas measurements. Our study specifically addresses knowledge gaps regarding the key microbial taxa and CA classes that drive soil CA activity and provides new constraints for models representing the influence of soils on atmospheric COS and CO^18^O.

## Materials and methods

### Soil collection and lab incubations

Soil samples were collected in triplicate within a 1-m sampling radius from the uppermost 10 cm (litter excluded) at 20 sites (Table S1). We sieved replicates separately and measured soil water holding capacity (WHC) and soil moisture. Soils were transferred in the amount of 80 g dry soil equivalent to sterilized 240-mL mason jars and soil moisture was adjusted to 30% WHC for a 7-day pre-incubation at room temperature (22.5 °C) in the dark. Two sets of pre-incubations were prepared for each replicate to conduct gas exchange measurements separately for moist (biological and abiotic) and dry (abiotic) conditions. The first soil set was wetted to 30% WHC using water enriched in ^18^O (δ^18^O–H_2_O = 47.57 ± 0.03‰ VSMOW), pre-incubated for 7 days in the dark, and then net COS and CO^18^O exchange rates were measured, which for COS represent the combination of simultaneous biological uptake and abiotic production. Directly following gas exchange measurements, we subsampled these moist soils to preserve for DNA (flash-frozen in liquid nitrogen), RNA (LifeGuard^®^ Soil Preservation Solution, MO BIO Laboratories, San Diego, CA, USA), and soil physical and chemical (Supplementary Information) analyses. The second soil set was wetted to 30% WHC with sterile water whose isotopic composition was unaltered, pre-incubated for 7 days, and then air-dried for a median of 45 days before measuring dry soil net COS exchange rates, which represent abiotic OCS production.

### Trace gas exchange measurements

All soils were transferred from pre-incubation jars to 1-L PFA chambers (100-1000-01, Savillex, Eden Prairie, MN, USA) to settle for 24 h before gas exchange measurements. The PFA chambers were installed on a dynamic flow-through chamber soil flux system described in Whelan et al. [27] for COS exchange measurements at 20 °C. Gas exchange was determined from the differences in COS, CO_2_, and H_2_O mole fractions measured in chamber outlet and inlet air flowing at ~0.3 L min^−1^ using a quantum cascade laser spectrometer (QCL, Aerodyne Research, Inc., Billerica, MA, USA). Mole fractions were measured during three cycles of a 40-min program for each soil replicate: inlet flow (10 min; measured with chamber bypass line), N_2_ tank (10 min; for QCL zeroing), and outlet flow (20 min; representing chamber air). For soils measured dry, room air was used as inlet air and only COS, CO_2_, and H_2_O mole fractions were quantified. For soils measured at 30% WHC, the rate of oxygen isotope exchange between CO_2_ in the chamber inlet air and soil water was also determined from inlet and outlet δ^18^O-CO_2_. For the dynamic chamber measurements at 30% WHC, inlet air was humidified and its composition was set with a mass flow control system to deliver mole fractions of CO_2_ and COS at ~450 parts per million and (ppm; e^−6^) and 450 parts per trillion (ppt; e^−12^), respectively. Discrete samples for CO_2_ isotope analysis were collected through a septum sampling port on the QCL sample inlet line by withdrawing 25 mL of gas sample and injecting into 12-mL pre-evacuated glass vials. Directly after the dynamic exchange measurement, ~60 g_soil,dw_ of soil was transferred to equilibrium chambers (950 cm^3^ sealed mason jars) for 3 days at 20 °C and gas samples for CO_2_ isotope analysis were collected through a septum port in the lids after 1, 2, and 3 days of equilibration. Vials were shipped to Forschungszentrum Jülich, Germany for analysis of both δ^18^O-CO_2_ and δ^13^C-CO_2_ with a continuous-flow isotope-ratio mass spectrometer (CF-IRMS, IsoPrime 100, Elementar Analysensysteme, Langenselbold, Germany) coupled with a TraceGas unit (Elementar Analysensysteme) for pre-concentration of sample gas. We report isotope ratios using delta notation (δ) using Vienna Standard Mean Ocean Water (VSMOW) and Vienna Pee Dee Belemnite (VPDB-CO_2_) as references for water and CO_2_, respectively.

### Data processing and gas exchange calculations

All analyses were done in R 3.1.10 (R Core Team, 2014) except for the multivariate analysis (Partial Least Squares, PLS, regression) performed using SIMCA^®^ (version 13.0.3.0, Umetrics™, Umeå, Sweden). Following pre-processing of QCL data (Supplementary Information), the exchange rate (*F*) of COS and CO_2_ was calculated from the difference between outlet (*c*_*o*_) and inlet (*c*_*i*_) mole fractions. For example, the net soil exchange rate for COS was calculated as:

1 $$F_{COS} = \frac{u}{S}\left( {c_{o,{\rm {COS}}} - c_{i,{\rm{COS}}}} \right)$$

where *u* (mol s^−1^) is the flow rate and *S* (0.0078 m^2^) is the soil surface area. Following others [27], we assumed the net soil exchange rate for COS from air-dried soils, which were always emissions of COS, well approximated the COS production rates at 30% WHC. We used this dry-soil production rate to partition net COS exchange measured in 30% WHC soils to determine COS consumption using *F*_COS_ = *F*_COS,production_+*F*_COS,consumption_ with *F*_COS,production_=*F*_COS,dry_. Additional discussion of the COS source term is given in Supplementary Information.

The fraction of CO_2_ flowing through the chamber that fully equilibrated with soil water (*f*_eq_) was determined from the isotopic composition of CO_2_ measured at the dynamic chamber inlet and outlet (δ_i_ and δ_o_, respectively) and inside the sealed equilibration chamber (δ_eq_) [25]:

2 $$f_{eq} = \frac{{C_o\delta _o - C_i\delta _i - \left( {C_o - C_i} \right)\delta _{eq}}}{{C_i\left( {\delta _{eq} - \delta _i} \right)}}$$

This formulation accounted for the contributions of respired CO_2_ already equilibrated with soil water as described in more detail in Supplementary Information.

### Model framework for deriving CA activity in soils

A gas transport model was used to represent COS and CO^18^O gas fluxes as the sum of three processes occurring simultaneously within the soil matrix: production, diffusion and a first-order enzymatic reaction. The model was applied to derive the CA-catalyzed rates for COS hydrolysis (*k*_COS_, s^−1^) from *F*_COS_ data following [46] and the CA-catalyzed rate for CO_2_-H_2_O isotopic exchange (*k*_CO2_, s^−1^) from measurements of *F*_CO2_, δ^18^O_F_, δ^18^O_a_ and δ^18^O_eq_ as in ref. [44]. A detailed description of this model is given in Supplementary Information.

### Sequencing and bioinformatics

DNA was extracted from each replicate (PowerSoil^®^ DNA Isolation Kit, MO BIO Laboratories, San Diego, CA, USA) and RNA was extracted from one replicate of ten sites (PowerSoil^®^ RNA Isolation Kit, MO BIO) from soils that were preserved directly after gas exchange measurements on moist soils. Phylogenetic amplicon iTag DNA sequencing from DNA extract with 16S rRNA (V4) and fungal ITS2 (ITS9F/ITS4R) primers and metatranscriptome sequencing from soil RNA were performed by the Department of Energy Joint Genome Institute (JGI), Walnut Creek, CA, USA. Data sets are available in JGI Genome Portal (https://genome.jgi.doe.gov/) under JGI proposal ID 2033. OTU tables were rarified to 40,000 and 80,000 sequences (GUniFrac R package [47]) and community composition was visualized using non-metric multidimensional scaling (NMDS, metaMDS, Bray–Curtis dissimilarity [48]). Spearman correlations between OTUs and environmental variables were considered robust for coefficients (ρ) > |0.5| and AdjPValue (q) < 0.01. Community diversity and richness metrics were calculated using alpha diversity estimates in Qiime v 1.9.1 and we report richness and diversity using the Observed OTUs and Shannon (H’) index, respectively [49].

We generated a comprehensive dataset encompassing alpha (COG3338), beta (COG0288), and gamma (COG0663) carbonic anhydrase gene classes from approximately 22,000 bacterial, archaeal, and eukaryote genomes from IMG/MER [50] and Mycocosm [51] in April, 2018. All genes were combined and clustered at 30% sequence similarity using Uclust [52], which resulted in 1361 clusters, centroids, and respective gene alignments. Hidden Markov Models (HMMs) were generated using 81 clusters that included at least 50 sequences. In total 80% of the sequences (retrieved using subsample_fasta.py in QIIME v. 1.9.1) in each cluster were used to generate the HMM using hmmbuild in HMMER version 3.1b2 [http://hmmer.org/]. Testing of each of the 81 HMMs was performed using hmmsearch in HMMER version 3.1.b2 [http://hmmer.org] on a mix of the remaining 20% of the sequences in each cluster (retrieved using filterbyname.sh in BBTools v. 37.76 [https://jgi.doe.gov/data-and-tools/bbtools/bb-tools-user-guide/bbmap-guide/]), which served as true positives, and reference sequences from all CA classes (alpha, beta, gamma, delta, eta, zeta). E-values and scores were determined based on entire gene sequences. HMMs were then searched against our soil metatranscriptomes using corresponding thresholds, and protein sequences were assigned to the cluster yielding the smallest E-values. Alignment and phylogenetic tree building of centroids from 81 clusters was performed using Clustal Omega [53]. Fast, scalable generation of high-quality protein multiple sequence alignments using Clustal Omega. Reads from each library were aligned to each of the reference transcriptomes (hits.fna) using BBMap in BBTools v. 37.76 [https://jgi.doe.gov/data-and-tools/bbtools/bb-tools-user-guide/bbmap-guide/] (BAMs/ directory) with only unique mapping allowed (parameters: ambig=toss strictmaxindel=4 minid=0.9). If a read mapped to more than one location, it was ignored. raw and fpkm normalized gene counts were generated using BBMap in BBTools v. 37.76 [https://jgi.doe.gov/data-and-tools/bbtools/bb-tools-user-guide/bbmap-guide/], which we expressed as CA transcripts per million (tpm) by normalizing by gene length and to 1,000,000 transcripts. We calculated correlations (Pearson) between the total tpm within CA classes and/or clades with CA-catalyzed reaction rates, excluding genomes <0.2 Gbp from the analysis because of limited CA recovery.

## Results

### Soil COS and CO^18^O exchange rates varied with biome and land use

**Fig. 1:**
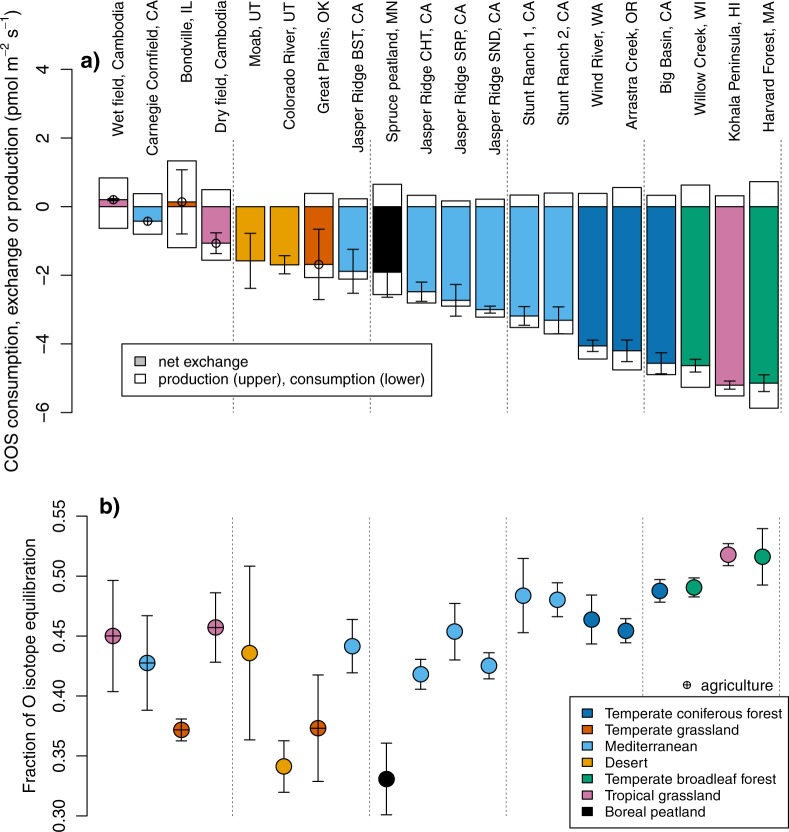
Variation in soil exchange rates of COS and CO^18^O with biome and land use. **a** COS net soil exchange (colored bars) and partitioned contributions of COS production (white bar upper) and consumption (white bar lower). **b** Fraction of atmospheric CO_2_ molecules that equilibrated oxygen isotopes with soil water within a 3-min residence time of the dynamic chamber measurement. Whiskers represent SD. Biome indicated by color key and agricultural sites denoted by ^⊕^ symbol

For a set of twenty sites encompassing a range of biomes and land use histories (Table S1), the net exchange of COS and the fraction of CO_2_ that attained isotopic equilibrium with soil water (*f*_eq_) were measured in soil microcosms. Biome and land use influenced the rates of net COS and CO_2_-H_2_O oxygen isotope exchange (Fig. 1 colored bars and points; Table S2), which were generally highest in forests and lower in Mediterranean ecosystems and deserts. Net exchange rates were lowest and most variable in soils from agricultural sites (crossed circles in Fig. 1). The net COS exchange represents a balance between enzymatic consumption of COS and putative abiotic COS production [27, 54]. We estimated COS production rates by measuring COS emissions from soils where biological COS consumption was limited by air drying [27, 45]. All air-dried soils emitted COS except those from deserts (Fig. 1a, top of white bars), and the highest emissions were measured in agricultural soils. Partitioning to distinguish COS consumption rates (Fig. 1a, bottom of white bars) revealed that the net COS exchange from agricultural soils was low both because of high COS production and low COS consumption rates compared to other ecosystems.

### Soil CA activity for COS and CO^18^O diverged with biome and land use

We used soil COS consumption and CO_2_-H_2_O isotopic equilibration to derive enzyme-catalyzed rates for both substrates using a trace gas model that describes the production, diffusion, and first-order consumption reactions for COS and CO^18^O in the soil matrix [46, 55]. The first-order soil reaction rates for CO_2_-H_2_O isotopic exchange during CO_2_ hydration (*k*_CO2_; s^−1^) and COS hydrolysis (*k*_COS_; s^−1^) were correlated (Fig. S1b; *r* *=* 0.55, *p* < 0.001) as were COS consumption and *f*_eq_ (Fig. S1a; *r* *=* 0.67, *p* *<* 0.001). This suggests that abiotic processes (i.e., diffusion limitation and un-catalyzed reaction rates) were not the primary drivers of trends in COS consumption and CO_2_ hydration (Fig. 1). Instead, biological activity was the main driver, and enzyme-catalyzed reaction rates were greater than uncatalyzed rates (Table 1). While absolute reaction rates were larger for CO_2_ than COS, the relative enhancement due to biological catalysis (*f*_*CA*_) was 2–3 orders of magnitude greater for COS (Table 1). Catalyzed reaction rates varied with biome and land use, designations that differentiate sites based on many factors including climate variables (Fig. S2). The ratio of COS to CO_2_ catalyzed reaction rates (*k*_COS_/*k*_CO2_) varied with biome and land use and was highest in forest and tropical grassland soils (0.77–0.84), intermediate in Mediterranean (predominantly grasslands with nearby oak scrub woodlands) and desert biomes (0.30–0.42), and lowest (0.20) in agricultural soils (Tables 1 and S2). CA activity did not consistently vary for COS and CO_2_, and their divergence was predominantly driven by variability in *k*_COS_ (Table 1). The divergence of catalyzed reaction rates for COS and CO_2_ could arise if different sets of taxonomic groups or CA classes dominate these two reactions in soils.

**Table 1:** Biome average and SD of CA reaction rates (*k*) and enhancement factors (*f*_*CA*_) for COS and CO_2_

Biome	n	*k*_*COS*_ (s^−1^)		*k*_*CO2*_ (s^−1^)		*k*_*COS*_ */k*_*CO2*_ *catalyzed*	*f*_*CA,COS*_	*f*_*CA,CO2*_
		uncatalyzed	catalyzed	uncatalyzed	catalyzed			
Tropical grassland	1	1.2 × 10^−5^	0.54	2.6 × 10^−3^	0.64	0.84	45000	110
Temperate coniferous forest	3	1.2 ± 0.006 × 10^−5^	0.38 ± 0.04	4.1 ± 0.8 × 10^−3^	0.49 ± 0.08	0.77 ± 0.09	32000 ± 3100	87 ± 14
Temperate broadleaf forest	2	1.2 ± 0.004 × 10^−5^	0.38 ± 0.02	5.2 ± 0.7 × 10^−3^	0.46 ± 0.02	0.82 ± 0.03	32000 ± 1800	82 ± 3
Mediterranean grassland	6	1.3 ± 0.1 × 10^−5^	0.20 ± 0.11	1.2 ± 1.0 × 10^−3^	0.48 ± 0.22	0.42 ± 0.24	17000 ± 9000	85 ± 38
Desert	2	21 ± 9.1 × 10^−5^	0.16 ± 0.06	0.034 ± 0.004 × 10^−3^	0.53 ± 0.06	0.30 ± 0.09	13000 ± 5400	93 ± 11
Agricultural	5	1.5 ± 0.8 × 10^−5^	0.08 ± 0.08	4.4 ± 2.4 × 10^−3^	0.38 ± 0.11	0.20 ± 0.14	6500 ± 6900	68 ± 20
Boreal peatland	1	1.2 × 10^–^^5^	0.04	5.8 × 10^−3^	0.08	0.56	3700	15

### Soil properties and microbial taxa as predictors of CA activity

**Fig. 2:**
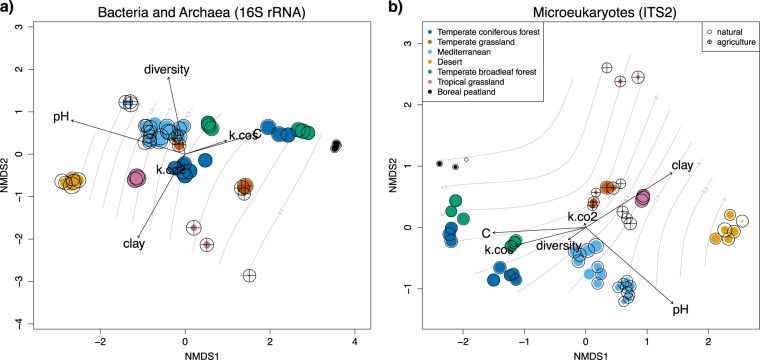
Soil microbial communities cluster by biome and align with soil pH, clay fraction, carbon, and diversity. Microbial communities of **a** bacteria and archaea and **b** eukaryotes shown using non-metric multidimensional scaling (NMDS) analysis (metaMDS) of Bray dissimilarity indices (Vegan) of rarified (GUniFrac) OTU tables. Point size reflects the model-derived soil CA reaction rates, *k*_*COS*_ (color-filled) and *k*_*CO2*_ (open circles), on a log-scale. Relationships between microbial community structure, edaphic factors, and catalyzed reaction rates given by arrows (direction of increasing gradient, length scaled to correlation; envfit); gray contours show soil pH gradient

We sequenced soil communities of bacteria and archaea (16 S rRNA gene amplicon sequencing) and microeukaryotes including fungi, algae, and protozoa (ITS2 amplicon sequencing) (Fig. S3) and calculated richness and diversity metrics. Of the measured soil properties (Table S3-S5), microbial diversity varied most strongly with soil C/N (*r* *=* 0.44, *p* *<* 0.001 and *r* *=* 0.53, *p* *<* 0.001 for correlations with bacteria/archaea and microeukaryotes, respectively). As in some previous studies [56], the diversity of the bacterial and archaeal communities increased with soil pH (*r* *=* 0.62, *p* *<* 0.001), but only when excluding desert soils (high pH, low diversity). Soil microbial community composition typically clustered by biome, and differences in composition was aligned with differences in soil pH, texture, nutrients, and microbial diversity (Fig. 2). Microbial communities from agricultural soils (Fig. 2 crossed circles) and other soils with divergent CA activity for COS and CO_2_ (Fig. 2 represented by white space between open and closed circles) were more distinct.

We used partial least squares (PLS) regression to assess the ability of 25 physical, chemical, and ecological soil properties to predict CA activity and its divergence for COS and CO_2_. The multivariate models explained 85%, 42%, and 79% of the variance in *k*_COS_, *k*_CO2_, and their ratio (*k*_COS_/*k*_CO2_), respectively (Fig. S4a-c). Dominant predictors of CA activity were structural (bulk density and texture) and chemical (e.g., carbon, nitrogen, phosphorus) soil properties, consistent with previous work showing correlations of soil CA activity with soil organic matter, total nitrogen, and C/N ratio [35]. Soils with lower levels of divergence in CA activity for COS and CO_2_ (larger *k*_COS_/*k*_CO2_) were associated with higher levels of soil carbon and nitrogen, coarser texture, lower bulk density, higher microeukaryote richness and diversity, and lower richness and diversity of bacteria and archaea (Fig. S4a-c and Table S5). Thus, CA activity for COS may be particularly sensitive to changes in soil nutrient levels and soil structure. Our agricultural soils were relatively high in clay and low in microbial biomass, carbon, and nitrogen (Fig. S5 and Tables S3-S5). Models of agricultural soils explained less total variance than models of non-agricultural soils (68% versus 83% of *k*_COS_/*k*_CO2_; 56 versus 90% of *k*_COS_, respectively), except for *k*_CO2_ (57% in agricultural; not significant for non-agricultural) and identified different predictors (Fig. S4d-e). We found that the divergence in CA activity for the two tracers was related to covariations in soil properties and microbial communities across the gradient of biome and land use. The observed co-variations of soil properties, microbial communities, and CA activity may be useful for empirical modeling of soil CA activity.

We investigated whether specific taxonomic groups dominated the correlation between CA activity and community-wide diversity metrics. We found that *k*_COS_ was positively correlated with the relative abundance of 19 operational taxonomic units (OTUs) from the fungal lineages *Ascomycota*, *Basidiomycota*, and *Zygomycota*, but only 2 OTUs (α-*Proteobacteria*) from bacterial lineages (Table S7). *k*_COS_/*k*_CO2_ was positively correlated with 41 fungal OTUs (predominantly *Ascomycota* from the *Leotiomycetes* class but also *Basidiomycota* and *Zygomycota*) but only 3 bacterial OTUs. In contrast, *k*_COS_/*k*_CO2_ was negatively correlated with 18 bacterial OTUs, 2 green algae OTUs (*Chlorophyta*), and only 4 fungal OTUs (Table S7), and trends were similar at the phylum level (Table S8). These results suggest a role for fungi in COS consumption and for algae and bacteria in CO_2_ exchange.

### Patterns in soil CA expression were related to soil CA activity

**Fig. 3:**
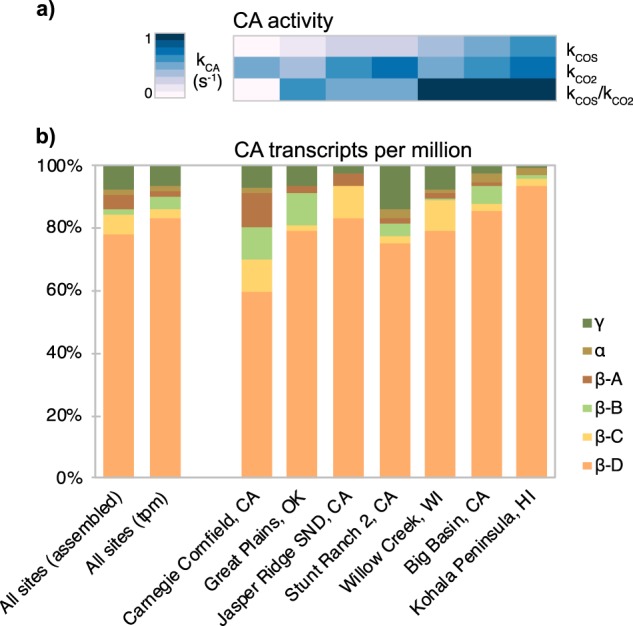
Patterns in measured CA activity in soils in relation to the diversity of expressed CA. **a** Enzyme-catalyzed reaction rates for COS (*k*_*COS*_) and for CO_2_ (*k*_*CO2*_) and their ratio (*k*_*COS*_/*k*_*CO2*_) were derived from gas flux measurements for comparison to gene expression pattern. **b** β-CA were the most highly expressed class of CA in soil, both in terms the relative abundance of assembled CA and mapped reads (transcripts per million, *tpm*) in all sites (7 metatranscriptomes). Within the β-CA class, clade D was the most highly expressed in all soils, with other CA types having a greater influence in the agricultural soils (Great Plains, OK and Carnegie Cornfield, CA)

We sequenced soil metatranscriptomes to evaluate whether patterns in soil CA gene expression underlie concurrent differences in CA activity for COS and CO_2_. We built custom Hidden Markov Models (HMMs) from the major clusters of equivalent CA diversity found in genome databases (Fig. S6). Using these HMMs, we recovered assembled CA genes from ten soil metatranscriptomes. Three CA classes (α, β, and γ) were found in our soils (Table S9), whereas the other three CA classes (δ, η, and ζ) that tend to have narrow phylogenetic distributions [4, 36, 39] were not found in appreciable abundance. We represented CA expression levels as the relative tpm of raw transcript reads mapped to assembled CA. Soil CA diversity was dominated by β-CAs, with low relative abundance of γ-CAs and α-CAs both in terms of assembled CA and number of reads (Fig. 3). CAs exhibit sequence diversity that can be organized within distinct clades (e.g., clades A-D for β-CAs) [4]. Soil CA expression was dominated by β-CA from clade D (Fig. 3b), and particularly β-CA HMM (Fig. S7) that contain CA from *Ascomycota* and *Basidiomycota CA* (clusters 129 and 11), and also from *Actinobacteria* (cluster 207), and *Proteobacteria* (cluster 6) reference genomes (Fig. S8). The most expressed gamma CA (clusters 15 and 628) suggest that *Actinobacteria* were the dominant source of γ-CA expression in soils (Fig. S7). α-CA expression (clusters 145 and 154) was associated to *Proteobacteria*. CA expression was unevenly distributed across both CA diversity and soil taxa indicating that soil CA profiles depend on the relative abundance of CA-expressing members of the soil community.

Soil CA expression levels were further analyzed to determine whether a particular class of CA or taxonomic group expressing CA were predictive of trends in COS and CO_2_ catalyzed reaction rates. Trends in *k*_COS_ (Fig. 3a) were correlated with CA expression levels (*tpm*; Fig. 3b) of β-CA from clade D (*r* = 0.83, *p* < 0.02), while β-A-CA *tpm* and *k*_COS_ were anti-correlated (*r* *=* −0.94, *p* *<* 0.01), and relationships with clades B and C were not significant. Patterns similar to those between *k*_*COS*_ and β-CA were observed for *k*_*CO2*_, but lacked significance. The relationship of *k*_CO2_ with β-D-CA *tpm* was weak (*r* = 0.35, *p* > 0.05), and instead the strongest relationship for *k*_CO2_ was with α-CA *tpm* (*r* = 0.53, *p* > 0.05). Trends with α-CA and γ-CA and were difficult to discern given their lower recovery rates compared to β-CA (Table S9). These results suggest that the CA enzyme pools may partially overlap for COS and CO_2_, but not completely.

## Discussion

### Soil CA activity depended on the intra- and inter-class diversity of CA

We propose that the divergence in soil CA activity for COS and CO^18^O depends on CA diversity because CA intrinsic properties (i.e., *k*_max_/*K*_m_) vary with CA class differently for COS and CO_2_. Significantly greater rates of COS catalysis are described for β-CA (1 s^−1^ μM^−1^) than α-CA (0.001 s^−1^ μM^−1^) [37]. In contrast, differences in CA activity for CO_2_ are relatively small, with α-CA typically having only slightly higher *k*_cat_/*K*_m_ (100 s^−1^ μM^−1^) than β-CA (10 s^−1^ μM^−1^) and γ-CA (10 s^−1^ μM^−1^) [57, 58]. We reason that COS hydrolysis in soils was driven by β-CA because β-CA expression levels and COS affinity are high. Our results suggest that this trend may be predominantly attributed to the dominant β-CA clade D, however intra-class variations in β-CA kinetic parameters are not well known. Carbonyl sulfide hydrolase (COSase) is a β-D-CA described in *Thiobacillus thioparus* with high affinity for COS and low specificity for CO_2_ [37]. *Thiobacillus* spp. OTU were rare in our soils and CA with characteristic COSase amino acid residues were not found. However, the *T. thioparus* COSase sequence grouped in β-D-CA clusters found in highest abundance in soils and that were predictive of COS consumption in soils (Fig. S6). The significance of γ-CA activity for COS is unclear because, to our knowledge, its intrinsic activity for COS has not been determined, but would have to compensate for the relatively low abundance of γ-CA to match the importance of β-CA. CO_2_ hydration may have been driven both by α-CA and β-CA, because of trade-offs between CO_2_ affinity (higher for α-CA) and CA expression levels in soils (higher for β-CA). Models of soil CA activity could account for differences in CA kinetic parameters and their variation with biome and/or soil microbial community, though outside of this study, these differences are not yet well cataloged.

### Soil CA activity in key taxonomic groups

We detected coherence between soil CA activity for COS and β-CA expression by *Ascomycota*, *Basidiomycota*, and *Actinobacteria* (Fig. S7 and S8), some of which are known to consume COS [37]. Fungal genomes are often under-represented in genomic databases, but we included >2000 fungal CA genes (α and β) when constructing our HMMs and found CA expression in clusters associated with fungal β-CA, but not α-CA consistent with the understanding that *Ascomycota* encode for β-CA at a higher genome frequency than α-CA [59]. While the role of fungi inferred in this study could be affected by fITS9/ITS4 primer biases that over-represent *Ascomycota* at the expense of *Basidiomycota* [60], the observation that fungi are predictive of soil CA activity for COS was further supported by correlations of *k*_COS_ with relative abundance of *Ascomycota* OTUs from classes with representatives previously shown to degrade COS (*Sordariomycetes* and *Leotiomycetes*) [43] and with community-level fungal diversity metrics. Fungi emerge as important drivers of CA activity for COS in and are often less abundant and diverse in agricultural systems that can have low C:N and undergo tillage [61, 62], which may explain the particular sensitivity of COS to land use and our observed trends in *k*_*COS*_/*k*_*CO2*_ (Table 1 and S2). These results are consistent with the findings of [44] using phylogenetic marker gene quantitative PCR (qPCR) for fungi. CA activity for CO_2_ was instead more related to α-CA than β-CA expression. We observed α-CA expression in clusters associated with *Proteobacteria*, and not from other groups known to contain α-CA such as *Ascomycota* and *Firmicutes* (Fig. S8). The low recovery rates of α-CA and γ-CA made it much more difficult to assess statistical relationships and patterns with taxonomy compared to β-CA (Table S8). Algae such as *Chlamydomonas reinhardtii* are known to express α-CA at high levels under ambient CO_2_ concentrations [63] and enhanced CA activity for CO_2_ by phototrophs has been shown in soils incubated under light conditions using qPCR [44]. While we observed relationships between CA activity for CO_2_ and the abundance of algal OTUs, expression of algal CA would be difficult to discern because algae were not well represented in the major CA clusters, only a small fraction of total mRNA originates from microeukaryotes [64], and our soils were incubated in the dark. Our results therefore build our understanding of non-photosynthetic CA activity in soils.

### Relevance to knowledge gaps

Models that represent key physical and chemical factors affecting COS and CO^18^O exchange in soils can predict sensitivity to physical drivers (e.g., temperature, moisture) over a wide range of temporal scales (days to years) [13, 30, 46, 65]. However, no systematic method exists for estimating the biological factors (e.g., soil CA activity) driving differences in COS and CO^18^O fluxes. We used a soil functional genomics approach to address this knowledge gap under controlled laboratory conditions. We leveraged genome databases and developed tools to profile CA expression across soils to, for the first time, describe robust relationships between microbial taxonomy, CA genes, and enzyme kinetics implicated in COS and CO^18^O soil fluxes. These relationships suggest that soil CA activity may also be indirectly sensitive to environmental factors through their influence microbial community composition and activity, and this study provides ample hypotheses for future work. We propose that soil CA and its two trace gas substrates (COS and CO^18^O) represent a valuable gene-to-function model system for probing the role of soil microbial diversity on ecosystem function and atmospheric composition.

### Conclusions and future outlook

We evaluated the physical, chemical, and ecological drivers of soil trace gas exchange in a diverse set of soils and found that differences in microbial community composition and gene expression were associated with differences in soil CA activity for COS and CO_2_ (*k*_COS_ and *k*_CO2_). COS consumption was predominantly driven by β-CA (especially β-D-CA) from *Ascomycota*, *Basidiomycota*, *Actinobacteria*, and *Proteobacteria*. CO_2_ hydration was driven more by α-CA from *Proteobacteria* or algae, with contributions from *Actinobacteria* β-CA*. Ascomycota*, *Basidiomycota*, *Actinobacteria*, and *Proteobacteria* are ubiquitous and abundant members of the soil microbiome and may be important global drivers of CA activity in soils. Barriers to predicting soil exchange rates of COS and CO^18^O can be directly addressed with our findings. Soil gas exchange rates and CA activity varied with biome and land use and could be estimated from inventories. Soil CA activity for COS and CO^18^O were correlated, and soil CA activity measured for one tracer could be used to estimate the other with acknowledgement of patterns in divergence. We present empirical models of CA activity as a function of soil properties alone, and in combination with soil CA expression levels, which may be used to predict soil CA activity given appropriate soil property or genome databases. Finally, we identify key taxa and CA diversity to guide the selection of kinetic parameters to better model soil CA activity alongside other important factors (e.g., soil temperature, moisture) that influence COS and CO^18^O gas exchange rates in soil.

We suggest that future efforts to overcome barriers to predicting soil CA activity for COS and CO_2_ from sequence data focus on the following research areas: (1) Needed are new databases of the distribution of CA in organisms (especially microeukaryotes such as fungi and algae) to link microbial taxonomy to environmental CA and to identify isolates for kinetic and physiological studies. (2) The distribution of CA should be characterized in other soils and environments and over time with additional analyses of metagenomes and metatranscriptomes, development of primers for CA, focus on microeukaryote gene expression [64], and in depth analysis of the microbial populations active in soil such as using stable isotope probing techniques. (3) Measurements are needed of kinetic parameters for reactions of COS and CO_2_ with the different classes (e.g., γ-CA) and clades (β-CA clades) of CA as well as assessment of other enzymes that may consume (CS_2_ hydrolase, RuBisCO, CO dehydrogenase, and nitrogenase) and produce (e.g., thiocyanate hydrolase) [66] COS in soils. These advances should be combined with further development of soil trace gas models that represent the influence of environmental factors  to help constrain soil COS and CO^18^O gas fluxes over a variety of relevant spatial and temporal scales.

## Electronic supplementary material

Supplemental Tables and Figures

Supplemental Information
