# Supplementary material for: Soil exchange rates of COS and CO18O differ with the diversity of microbial communities and their carbonic anhydrase enzymes
Source: ISME J. 2018 Sep 13;13(2):290–300. doi: 10.1038/s41396-018-0270-2 (PMC6330096; doi:10.1038/s41396-018-0270-2)
Supplement: Supplementary file 1 — Supplemental Tables and Figures [file 41396_2018_270_MOESM1_ESM.pdf]

Table S1 Soil sampling site information. Table sorted by biome, and then alphabetically by site name.

Sample ID[a]	Location	GPS [b]	Biome [c]	Ecosystem and Site	Treatment history	Date collected	Related COS measurements
CM-DF	Dry Field, Cambodia	11°30'20.89"N, 105° 0'31.53"E	Tropical and subtropical moist broadleaf forests	Grazed tropical grassland, seasonally saturated wetland. Higher elevation, drier part of field. Drained at time of sampling. Snake Pointe site P Mekong River Delta.	Medium-impact grazing. Rice cultivated 5-10 years ago. Flooded 4-5 months per year. No current fertilizer application known. Low intensity cattle grazing.	5/20/2015	-
CA-WF	Wet Field Cambodia	11°30'20.89"N, 105° 0'31.53"E	Tropical and subtropical moist broadleaf forests	Grazed tropical grassland, seasonally saturated wetland. Lower elevation, wetter part of field. Few cm water at time of sampling. Snake Pointe site P Mekong River Delta,	Medium-impact grazing. Rice cultivated 5-10 years ago. Flooded 4-5 months per year. No current fertilizer application known. Low intensity cattle grazing.	5/20/2015	-
HI-KP	Kohala Peninsula, HI	20° 8'49.38"N, 155°50'3.40"W	Tropical and subtropical grassland savannas and shrublands	C4 grasses for pasture on leeward slope of Kohala volcano. ~150kya parent material. Intermediate precipitation site in sequence (6).	Grasslands used for low impact cattle grazing.	3/17/15	-
IL-BV	Bondville, IL	40° 0'22.32"N, 88°17'25.44"W	Temperate grasslands, savannas and shrublands	Soybean/corn. Fluxnet, US-Bo1	Continuous no-till agriculture, annual rotation between corn and soybeans. Sampled after harvest.	4/22/15	(1, 2)
OK-GP	Great Plains, OK	36°36'18.00"N, 97°29'6.00"W	Temperate grasslands, savannas and shrublands	Wheat field near fields used for pasture. Sampled near Lamont, Southern Great Plains ARM Central Facility, Oklahoma: EF13	Wheat field. Sampled before harvest.	5/19/15	(1–3)
OR-AC	Arrastra Creek, OR	42° 11'5.19"N, 122°47'45.82"W	Temperate coniferous forests	Open conifer forest on steep slope (ponderosa pine, douglas fir, white fir). Permit: BLM-ORM0002011-02	Mixed forest management. Recent thinning and possible selective thinning in the past	5/22/15	-
CA-BB	Big Basin, CA	37°11'35.56"N, 122°13'13.48"W	Temperate coniferous forests	Coastal redwood forest on slight slope	No significant use; old growth rainforest	4/24/15	-
WA-WR	Wind River, WA	45°49'13.80"N, 121°57'6.84"W	Temperate coniferous forests	Douglas-fir and western hemlock transitional overstory	Old growth forest	4/27/15	-
MA-HF	Harvard Forest, MA	42°32'12"N, 72°10'24"W	Temperate broadleaf and mixed forests	Hemlock, oak, beech, birch and maple forest with significant litter layer within Environmental Measurement Station flux tower footprint. RPA #3335	Mature secondary forest aged 80 to 115 years	5/21/15	(4, 5)
MN-SP	Spruce peatland, MN	47°30'10.26"N, 93°28'58.20"W	Temperate broadleaf and mixed forests	Picea mariana. bog dominated by mature Populus tremuloides Michx. and Betula papyrifera Marsh. Sphagnum and bryophytes. Marcell Experimental Forest, SPRUCE ORNL project, Minnesota	Primary bog and peatland boreal forest	4/9/15	-
WI-WC	Willow Creek, WI	45°48'20.34"N, 90° 4'46.13"W	Temperate broadleaf and mixed forests	Secondary stands of sugar maple-aspen-yellow birch forests. Relatively open forest, soils with broadleaf litter and grasses. US-WCr Fluxnet site.	Secondary forest following last clearcut in 1930s.	4/29/15	(1, 2)

CA-CC	Carnegie Cornfield, CA	37° 25'44.06"N, 122°10'48.12"W	Mediterranean forests, woodlands and shrublands	Cornfield in agricultural research field, bare during sampling. Carnegie Institution, Stanford campus.	Corn rotations, plowed the week before sampling. Fertilized with $(NH_4)_2SO_4$ each year; horse manure was spread a couple of years prior.	4/17/15	-
CA-JRB	Jasper Ridge BST, CA	37°24'17.32"N, 122°13'33.51"W	Mediterranean forests, woodlands and shrublands	Blue schist. Low productivity, shallow, poorly developed soils. Mix of exotic annuals and native forbs and grasses.	Native grassland in biological preserve	4/1/15	-
CA-JRC	Jasper Ridge CHT, CA	37°24'18.06"N, 122°13'37.07"W	Mediterranean forests, woodlands and shrublands	Chert parent material. Low productivity, shallow, poorly developed soils. Mix of exotic annuals and native forbs and grasses.	Native grassland in biological preserve	4/1/15	-
CA-JRSN	Jasper Ridge SND, CA	37°24'24.63"N, 122°13'40.08"W	Mediterranean forests, woodlands and shrublands	Sandstone parent material. Moderately productive, well-developed soils. Dominated by exotic European annual grasses.	Native grassland in biological preserve	4/1/15	-
CA-JRSR	Jasper Ridge SRP, CA	37°24'21.70"N, 122°13'37.08"W	Mediterranean forests, woodlands and shrublands	Serpentine parent material Low productivity, shallow, poorly developed soils. Mix of exotic annuals and native forbs and grasses.	Native grassland in biological preserve	4/1/15	-
CA-SR1	Stunt Ranch 1, CA	34° 5'38"N, 118°39'26"W[b]	Mediterranean forests, woodlands and shrublands	Oak savannah (*Quercus agrifolia*). 4.5 m from oak tree. Stunt Ranch Reserve, Soil chamber 1	Primary coast live oak woodland, and annual grasslands.	4/24/15	(2, 7, 8)
CA-SR2	Stunt Ranch 2, CA	34° 5'38"N, 118°39'26"W[b]	Mediterranean forests, woodlands and shrublands	Oak savannah (*Quercus agrifolia*). Beneath oak tree. Stunt Ranch Reserve, Soil chamber 2	Primary coast live oak woodland, and annual grasslands.	4/24/15	(2, 7, 8)
UT-CR	Colorado River, UT	38º40'58"N, 109º25'21"W[b]	Desert and xeric shrublands	Shrubland, red desert, river gorge. Low shrubs and some grass at a distance of at least 1 m.	No significant use; desert lands.	3/28/15	-
UT-MB	Moab, UT	38º52'4"N, 109º48'48"W[b]	Desert and xeric shrublands	Shrubland, gray desert, flat. Some grasses and herbs, cattle droppings. Sampled 3-5 m from nearest shrub	Evidence of low intensity cattle grazing; desert lands.	3/28/15	-

[a]Site code from Meredith, L.K.; Boye, K.; Youngerman, C.; Whelan, M.; Ogée, J.; Sauze, J.; Wingate, L. Coupled Biological and Abiotic Mechanisms Driving Carbonyl Sulfide Production in Soils. *Soil Syst.* **2018**, *2*, 37.
[b]iPhone GPS projection
[c]Biome map (9)

1. Montzka S a., et al. (2007) On the global distribution, seasonality, and budget of atmospheric carbonyl sulfide (COS) and some similarities to CO2. *J Geophys Res Atmos* 112:1–15.
2. Whelan ME, et al. (2016) Carbonyl sulfide exchange in soils for better estimates of ecosystem carbon uptake. *Atmos Chem Phys* 16:3711–3726. Available at: http://www.atmos-chem-phys-discuss.net/15/21095/2015/.
3. Maseyk K, et al. (2014) Sources and sinks of carbonyl sulfide in an agricultural field in the Southern Great Plains. *Proc Natl Acad Sci U S A* 111:9064–9. Available at: http://www.ncbi.nlm.nih.gov/pubmed/24927594.
4. Commane R, et al. (2015) Seasonal fluxes of carbonyl sulfide in a mid-latitude forest. *PNAS* 112(46):14162–14167.
5. Mellillo JM, Steudler PA (1989) The effect of nitrogen fertilization on the COS and CS_2 emissions from temperature forest soils. *J Atmos Chem* 9(4):411–417. Available at:

http://dx.doi.org/10.1007/BF00139075%5Cnhttp://springerlink.metapress.com/openurl.asp?genre=article&id=doi:10.1007/BF00114753.
6. Austin AT, Vitousek PM (1998) Nutrient dynamics on a rainfall gradient in Hawai'i. *Oecologia* 113(4):519–529.
7. Sun W, Maseyk K, Lett C, Seibt U (2016) Litter dominates surface fluxes of carbonyl sulfide in a Californian oak woodland. *J Geophys Res G Biogeosciences* 121(2):438–450.
8. Sun W, Maseyk K, Lett C, Seibt U (2015) A soil diffusion–reaction model for surface COS flux: COSSM v1. *Geosci Model Dev* 8(10):3055–3070. Available at: http://www.geosci-model-dev.net/8/3055/2015/.
9. Olson DM, et al. (2001) Terrestrial Ecoregions of the World: A New Map of Life on Earth. *Bioscience* 51(11):933. Available at: https://academic.oup.com/bioscience/article/51/11/933-938/227116.

Table S2 Biome differences for a series of response values. Similar letters within a column mean no statistical difference (α = 0.05) between biomes. The effect of biome (here "biome" is a combination of "Biome" and "Land use"; for example, Temperate Evergreen Forest) on ten response variables, was evaluated with linear mixed models and ANOVAs. All models used Site ID as a random effect. Least squared means (R package lsmeans) was used to determine the difference in mean response variable values between biomes. Significant differences of means ($p \leq 0.05$) were determined using Tukey's adjustment. Biome was not a significant predictor of the response variables $k_{CO2,uncatalyzed}$ and $k_{CO2,catalyzed}$. Biome was a significant predictor of all other response variables ($p < 0.001$).

	Lsmeans							
biome	F_{COS}	F_{COS} consumption	f_{eq}	$k_{COS,uncatalyzed}$*	$k_{COS,catalyzed}$	k_{COS}/k_{CO2} catalyzed	$f_{CA.COS}$*	$f_{CA.CO2}$
Tropical grassland	-5.20 A	-5.52 A	0.52 B	1.2E-5 A	0.54 D	0.85 B	45000 B	110 B
Temperate coniferous forest	-4.27 A	-4.70 A	0.47 AB	1.2E-5 A	0.38 CD	0.78 B	32000 B	86 B
Temperate broadleaf forest	-4.89 A	-5.57 A	0.50 B	1.2E-5 A	0.38 BCD	0.83 B	32000 B	82 B
Mediterranean grassland	-2.77 B	-3.04 B	0.45 AB	1.3E-5 A	0.20 ABC	0.42 A	15000 AB	78 B
Desert	-1.64 BC	-1.58 C	0.39 AB	2.0E-4 B	0.16 ABC	0.33 A	12000 AB	89 B
Agricultural	-0.57 C	-1.26 C	0.41 AB	1.4E-5 A	0.08 A	0.20 A	4300 A	65 B
Boreal peatland	-1.91 BC	-2.56 BC	0.33 A	1.2E-5 A	0.04 AB	0.53 AB	3200 AB	14 A

*Data for model were log transformed for analysis and back-transformed for presentation.

Table S3 Physical properties of soils averaged for 3 replicates. Table sorted by land and biome, followed by site name.

Site Name	Land Use/Biome	Clay (%)	Silt (%)	Sand (%)	WHC (g,H2O/g,soil)	Soil, dry (g)	GWC (g,H2O/g,soil)	VWC (v,H2O/v,soil)	BD, dry (g/cm3)	pH
Kohala Peninsula, HI	TGS	4	30	66	0.84	79	0.26	0.17	0.84	6.5
Arrastra Creek, OR	TCF	3	28	69	0.86	80	0.25	0.15	0.70	6.1
Big Basin, CA	TCF	5	40	55	0.78	79	0.24	0.15	0.77	6.3
Wind River, WA	TCF	1	5	95	0.73	83	0.17	0.12	0.77	5.7
Harvard Forest, MA	TBF	2	28	70	1.61	79	0.49	0.18	0.59	4.6
Willow Creek, WI	TBF	2	16	82	0.88	77	0.31	0.19	0.79	5.8
Jasper Ridge BST, CA	MED	6	42	52	0.76	75	0.30	0.19	0.79	7.6
Jasper Ridge CHT, CA	MED	6	44	50	0.61	76	0.23	0.16	0.86	7.1
Jasper Ridge SND, CA	MED	9	56	35	0.49	75	0.19	0.14	0.92	6.8
Jasper Ridge SRP, CA	MED	5	48	47	0.83	73	0.34	0.19	0.80	7.6
Stunt Ranch 1, CA	MED	7	42	51	0.37	80	0.10	0.09	1.01	7.2
Stunt Ranch 2, CA	MED	4	37	59	0.66	82	0.17	0.12	0.87	6.4
Colorado River, UT	DES	7	41	51	0.23	79	0.08	0.09	1.32	9.2
Moab, UT	DES	19	76	6	0.43	77	0.16	0.13	0.93	9.5
Dry field, Cambodia*	AGR/TGS	15	75	10	0.69	75	0.27	0.19	0.88	5.5
Wet field, Cambodia*	AGR/TGS	13	70	16	0.81	69	0.42	0.36	0.67	4.5
Bondville, IL	AGR/TGL	4	40	56	0.61	80	0.23	0.16	0.83	5.4
Great Plains, OK	AGR/TGL	7	68	25	0.32	79	0.10	0.08	0.87	5.0
Carnegie Cornfield, CA	AGR/MED	7	50	44	0.51	76	0.21	0.17	0.92	8.4
Spruce Peatland, MN	BOR	n.d.	n.d.	n.d.	20.80	7	9.58	0.24	0.02	4.1

* 2 replicates
n.d. not determined

Table S4 Chemical and gas flux properties of soils averaged for 3 replicates. Table sorted by land use and biome, followed by site name.

Site Name	Land Use/Biome	C (%)	N (%)	C:N	P (%)	CO_2 flux (μmol m^{-2} min^{-1})	COS net flux (pmol m^{-2} min^{-1})	COS consumption (pmol m^{-2} min^{-1})	COS production (pmol m^{-2} min^{-1})	f_{eq}	k_{COS} (s^{-1})	k_{CO2} (s^{-1})
Kohala Peninsula, HI	TGS	4.81	0.47	10	0.634	12.9	-5.2	-5.5	0.3	0.52	0.54	0.64
Arrastra Creek, OR	TCF	6.49	0.20	32	0.071	26.6	-4.2	-4.8	0.6	0.45	0.34	0.40
Big Basin, CA	TCF	4.65	0.19	25	0.139	11.1	-4.6	-4.9	0.3	0.49	0.41	0.52
Wind River, WA	TCF	4.36	0.13	33	0.101	11.5	-4.1	-4.4	0.4	0.46	0.39	0.56
Harvard Forest, MA	TBF	8.10	0.38	21	0.098	19.3	-5.1	-5.9	0.7	0.52	0.39	0.45
Willow Creek, WI	TBF	4.75	0.32	15	0.102	19.5	-4.6	-5.3	0.6	0.49	0.36	0.48
Jasper Ridge BST, CA	MED	1.61	0.13	12	0.033	20.9	-1.9	-2.1	0.2	0.44	0.09	0.36
Jasper Ridge CHT, CA	MED	2.21	0.20	11	0.057	16.5	-2.5	-2.8	0.3	0.42	0.14	0.33
Jasper Ridge SND, CA	MED	1.44	0.13	11	0.020	13.0	-3.0	-3.2	0.2	0.43	0.20	0.39
Jasper Ridge SRP, CA	MED	2.65	0.22	12	0.036	15.4	-2.7	-2.9	0.2	0.45	0.14	0.34
Stunt Ranch 1, CA	MED	1.44	0.07	21	0.104	8.2	-3.2	-3.5	0.3	0.48	0.38	0.88
Stunt Ranch 2, CA	MED	3.83	0.22	17	0.115	10.5	-3.3	-3.7	0.4	0.48	0.27	0.59
Colorado River, UT	DES	2.13	0.02	88	0.052	-11.6	-1.7	-1.6	-0.1	0.34	0.20	0.49
Moab, UT	DES	1.66	0.01	172	0.090	-18.3	-1.6	-1.6	0.0	0.44	0.11	0.57
Dry field, Cambodia*	AGR/TGS	1.01	0.12	9	0.064	8.3	-1.1	-1.6	0.5	0.46	0.06	0.44
Wet field, Cambodia*	AGR/TGS	2.27	0.23	10	0.040	6.4	0.2	-0.6	0.8	0.45	0.02	0.28
Bondville, IL	AGR/TGL	2.22	0.21	11	0.040	5.1	0.1	-1.2	1.3	0.37	0.05	0.24
Great Plains, OK	AGR/TGL	1.21	0.12	10	0.013	4.8	-1.7	-2.1	0.4	0.37	0.22	0.51
Carnegie Cornfield, CA	AGR/MED	1.27	0.10	12	0.074	32.7	-0.4	-0.8	0.4	0.43	0.04	0.44
Spruce Peatland, MN	BOR	42.35	1.29	33	0.444	14.9	-1.9	-2.6	0.7	0.33	0.04	0.08

* 2 replicates

n.d. not determined

Table S5 Microbial properties of soils averaged for 3 replicates. Table sorted by land use and biome, followed by site name.

Site Name	Land Use/Biome	Microbial biomass C (ug/kg)	Microbial biomass N (ug/kg)	16S rRNA Richness	16S rRNA Diversity	ITS2 Richness	ITS2 Diversity
Kohala Peninsula, HI	TGS	248	28	1140	4.3	687	3.7
Arrastra Creek, OR	TCF	568	87	1112	4.8	884	4.4
Big Basin, CA	TCF	261	28	949	4.8	719	3.9
Wind River, WA	TCF	160	15	1867	4.4	410	3.0
Harvard Forest, MA	TBF	1030	135	1714	4.7	644	3.6
Willow Creek, WI	TBF	797	147	2946	5.2	640	3.9
Jasper Ridge BST, CA	MED	465	60	3905	6.2	672	4.4
Jasper Ridge CHT, CA	MED	460	71	4138	6.2	749	4.2
Jasper Ridge SND, CA	MED	356	50	3608	6.0	753	4.0
Jasper Ridge SRP, CA	MED	400	52	3683	6.0	695	3.9
Stunt Ranch 1, CA	MED	192	28	3500	6.0	678	3.7
Stunt Ranch 2, CA	MED	409	56	3616	6.3	600	3.3
Colorado River, UT	DES	39	2	1026	4.9	322	3.3
Moab, UT	DES	122	13	793	4.3	360	2.6
Dry field, Cambodia*	AGR/TGS	151	6	965	5.0	811	4.2
Wet field, Cambodia*	AGR/TGS	379	51	973	4.8	838	4.5
Bondville, IL	AGR/TGL	178	16	3027	5.7	645	3.6
Great Plains, OK	AGR/TGL	195	16	2474	5.3	668	3.9
Carnegie Cornfield, CA	AGR/MED	241	35	3241	6.1	641	3.2
Spruce Peatland, MN	BOR	9442	5328	1001	4.6	297	2.7

* 2 replicates
n.d. not determined

Table S6 Summary of PLS model results including cumulative explanatory coefficient (Y_{cum}) and number of significant components in parentheses and top positive (+) and negative (-) components listed in order of significance

	**All (20 sites)**		**All (no peat; 19 sites)**		**Non-agricultural (no peat; 14 sites)**		**Agricultural (5 sites)**	
	**Y_{cum}**	**Coefficients**	**Y_{cum}**	**Coefficients**	**Y_{cum}**	**Coefficients**	**Y_{cum}**	**Coefficients**
$F_{COS,consumption}$ **	0.78 (3)	(+) silt, clay, BD<br>(-) sand, f_{eq}, P	0.86 (3)	(+) silt, clay, BD<br>(-) C, sand, f_{eq}	0.92 (3)	(+) pH, clay, BD<br>(-) C, N, f_{eq}	0.50 (1)	(+) microbial N, P, Zn<br>(-) silt, ITS2 richness, 16S rRNA diversity
$V_{d,COS}$**	0.80 (3)	(+) f_{eq}, sand, soluble SO_4<br>(-) silt, clay, BD	0.83 (2)	(+) C, f_{eq}, N<br>(-) silt, BD, clay	0.93 (3)	(+) C, N, S<br>(-) pH, BD, clay	0.46 (1)	(+) silt, ITS2 richness, 16S rRNA diversity<br>(-) WHC, Zn, P
f_{eq}	0.61 (2)	(+) ITS2 richness, sand, soluble SO_4<br>(-) $F_{COS,consumption}$**, microbial N, silt	0.49 (1)	(+) N, S, WHC<br>(-) $F_{COS,consumption}$**, BD, silt	0.57 (1)	(+) N, S, WHC<br>(-) $F_{COS,consumption}$**, BD, pH	0.78 (2)	(+) Zn, K, P<br>(-)16S rRNA richness, sand, C
k_{COS}	0.75 (2)	(+) sand, k_{CO2}, soluble SO_4<br>(-) silt, clay, VWC	0.76 (2)	(+) C, sand, P<br>(-) silt, clay, 16S rRNA diversity	0.90 (3)	(+) C, sand, P<br>(-) silt, pH, clay	0.56 (1)	(+) k_{CO2}, silt, 16S rRNA richness<br>(-) Zn, WHC, P
k_{CO2}	0.56 (2)	(+) k_{COS}, BD, K<br>(-) WHC, microbial C, VWC	0.34 (1)	(+) k_{COS}, P, soluble SO_4<br>(-) VWC, R, 16S rRNA diversity	n.s.	n.s.	0.57 (1)	(+) k_{COS}, BD, silt<br>(-) C, N, WHC
k_{COS}/k_{CO2}	0.64 (2)	(+) sand, P, soluble SO_4<br>(-) silt, clay, 16S rRNA diversity	0.80 (3)	(+) C, sand, N<br>(-) silt, clay, BD	0.83 (3)	(+) C, N, sand<br>(-) pH, silt, clay	0.68 (2)	(+) silt, ITS2 diversity/richness<br>(-) P, Zn, VWC

*cumulative explanatory coefficient (Y_{cum})

** Sign of correlation is affected by sign for COS consumption, which is negative for $F_{COS,consumption}$ and positive for $V_{d,COS}$ (COS deposition velocity).

n.s. no significant components

Table S7 Spearman correlations of catalyzed reaction rates for COS (k_{COS}), CO_2 (k_{CO2}), and their ratio (k_{COS}/k_{CO2}) with genus-level OTU taxonomic ranks (Level 6) for OTUs with correlation coefficients > |0.5| and adjusted p-values < 0.01. Significant correlations indicated in yellow when positive and in in orange when negative. Data in this table are listed first by k_{CA} type (k_{COS}, k_{CO2}, k_{COS}/k_{CO2}), then by amplicon target (16S rRNA followed by ITS2), and finally sorted by correlation strength (positive correlations followed by absolute strength of negative correlations).

k_{CA}	Amplicon	Taxon	Correlation	Adjusted p-value
k_{COS}	16S rRNA	k__Bacteria;p__Proteobacteria;c__Alphaproteobacteria;o__Rhizobiales;f__Xanthobacteraceae;g__uncultured_2554	0.56	1.7E-03
k_{COS}	16S rRNA	k__Bacteria;p__Proteobacteria;c__Alphaproteobacteria;o__Rhodospirillales;f__JG37_AG_20;g__Unknown_Genus_1000711	0.53	2.5E-03
k_{COS}	16S rRNA	k__Bacteria;p__Proteobacteria;c__Deltaproteobacteria;o__Syntrophobacterales;f__Syntrophobacteraceae;Other	-0.55	1.7E-03
k_{COS}	16S rRNA	k__Bacteria;p__Cyanobacteria;c__Cyanobacteria;o__SubsectionIV;f__FamilyI_1566;Other	-0.55	1.7E-03
k_{COS}	16S rRNA	k__Bacteria;p__Hydrogenedentes;c__Unknown_Class_1002397;o__Unknown_Order_1002398;f__Unknown_Family_1002399;g__Unknown_Genus_1000570	-0.54	1.9E-03
k_{COS}	16S rRNA	k__Bacteria;p__Proteobacteria;c__Deltaproteobacteria;o__Desulfuromonadales;f__21f08;Other	-0.53	2.5E-03
k_{COS}	16S rRNA	k__Archaea;p__Euryarchaeota;c__Methanobacteria;o__Methanobacteriales;f__Methanobacteriaceae;g__Methanobacterium	-0.51	3.9E-03
k_{COS}	ITS2	p__Ascomycota;c__Sordariomycetes;o__Hypocreales;f__Cordycipitaceae;Other;Other	0.64	2.7E-05
k_{COS}	ITS2	p__Zygomycota;c__Unknown_27287;o__Mucorales;f__Cunninghamellaceae;g__Absidia;Other	0.64	2.7E-05
k_{COS}	ITS2	p__Ascomycota;c__Leotiomycetes;o__Helotiales;Other;Other;Other	0.57	7.8E-04
k_{COS}	ITS2	p__Basidiomycota;c__Microbotryomycetes;Other;Other;Other;Other	0.57	7.8E-04
k_{COS}	ITS2	p__Basidiomycota;c__Agaricomycetes;o__Thelephorales;f__Thelephoraceae;g__Tomentella;Other	0.56	7.8E-04
k_{COS}	ITS2	p__Ascomycota;c__Dothideomycetes;o__Venturiales;f__Venturiaceae;Other;Other	0.56	7.8E-04
k_{COS}	ITS2	p__Basidiomycota;c__Agaricomycetes;o__Agaricales;f__Mycenaceae;g__Mycena;Other	0.56	7.8E-04
k_{COS}	ITS2	p__Ascomycota;c__Leotiomycetes;o__Helotiales;f__Dermateaceae;g__Cryptosporiopsis;Other	0.56	7.8E-04
k_{COS}	ITS2	p__Ascomycota;c__Eurotiomycetes;o__Eurotiales;f__Trichocomaceae;g__Paecilomyces;Other	0.55	8.2E-04
k_{COS}	ITS2	p__Ascomycota;c__Sordariomycetes;o__Coniochaetales;f__Coniochaetaceae;Other;Other	0.54	1.4E-03
k_{COS}	ITS2	p__Ascomycota;c__Eurotiomycetes;o__Eurotiales;f__Trichocomaceae;g__Sagenomella;Other	0.54	1.6E-03
k_{COS}	ITS2	p__Ascomycota;c__Eurotiomycetes;o__Chaetothyriales;f__Herpotrichiellaceae;g__Unknown_22134;Other	0.53	2.2E-03
k_{COS}	ITS2	p__Ascomycota;c__Sordariomycetes;o__Hypocreales;f__Clavicipitaceae;g__Pochonia;Other	0.52	2.5E-03
k_{COS}	ITS2	p__Ascomycota;c__Leotiomycetes;o__Unknown_22440;f__Unknown_22441;Other;Other	0.52	2.7E-03

k_{COS}	ITS2	p__Ascomycota;c__Sordariomycetes;o__Hypocreales;f__Clavicipitaceae;Other;Other	0.52	2.7E-03
k_{COS}	ITS2	p__Ascomycota;c__Eurotiomycetes;o__Eurotiales;f__Trichocomaceae;g__Unknown_22199;Other	0.51	2.8E-03
k_{COS}	ITS2	p__Ascomycota;c__Leotiomycetes;o__Unknown_22438;f__Unknown_22439;g__Leohumicola;Other	0.50	4.2E-03
k_{COS}	ITS2	p__Ascomycota;c__Unknown_21606;Other;Other;Other;Other	0.50	4.2E-03
k_{COS}	ITS2	p__Chlorophyta;Other;Other;Other;Other;Other	-0.51	3.9E-03
k_{CO2}	16S rRNA	na	na	na
k_{CO2}	16S rRNA	na	na	na
k_{COS}/k_{CO2}	16S rRNA	k__Bacteria;p__Proteobacteria;c__Alphaproteobacteria;o__Rhizobiales;f__Xanthobacteraceae;g__Variibacter	0.64	8.9E-06
k_{COS}/k_{CO2}	16S rRNA	k__Bacteria;p__Acidobacteria;c__Acidobacteria;o__Subgroup_3;f__Unknown_Family_357;g__Candidatus_Solibacter	0.62	3.0E-05
k_{COS}/k_{CO2}	16S rRNA	k__Bacteria;p__Verrucomicrobia;c__Spartobacteria;o__Chthoniobacterales;f__Xiphinematobacteraceae;g__Candidatus_Xiphinematobacter	0.59	1.0E-04
k_{COS}/k_{CO2}	16S rRNA	k__Bacteria;p__Proteobacteria;c__Alphaproteobacteria;o__Rhizobiales;f__Xanthobacteraceae;g__uncultured_2554	0.51	2.1E-03
k_{COS}/k_{CO2}	16S rRNA	k__Bacteria;p__Proteobacteria;c__Alphaproteobacteria;o__Rhodospirillales;f__JG37_AG_20;g__Unknown_Genus_1000711	0.51	2.4E-03
k_{COS}/k_{CO2}	16S rRNA	k__Bacteria;p__Chloroflexi;c__Ktedonobacteria;o__C0119;f__Unknown_Family_1002272;Other	-0.68	2.3E-06
k_{COS}/k_{CO2}	16S rRNA	k__Bacteria;p__Planctomycetes;c__Phycisphaerae;Other;Other;Other	-0.65	8.8E-06
k_{COS}/k_{CO2}	16S rRNA	k__Bacteria;p__Firmicutes;c__Clostridia;o__Clostridiales;f__Clostridiaceae_1;g__Clostridium_sensu_stricto_1	-0.60	6.5E-05
k_{COS}/k_{CO2}	16S rRNA	k__Bacteria;p__Firmicutes;c__Clostridia;o__Clostridiales;f__Ruminococcaceae;g__uncultured_2071	-0.60	6.5E-05
k_{COS}/k_{CO2}	16S rRNA	k__Bacteria;p__Proteobacteria;c__Gammaproteobacteria;o__Chromatiales;Other;Other	-0.60	6.5E-05
k_{COS}/k_{CO2}	16S rRNA	k__Bacteria;p__Bacteroidetes;c__Sphingobacteriia;o__Sphingobacteriales;f__Chitinophagaceae;g__Flavisolibacter	-0.57	3.3E-04
k_{COS}/k_{CO2}	16S rRNA	k__Bacteria;p__Proteobacteria;c__Alphaproteobacteria;o__Sphingomonadales;f__Erythrobacteraceae;Other	-0.56	4.1E-04
k_{COS}/k_{CO2}	16S rRNA	k__Bacteria;p__Proteobacteria;c__Deltaproteobacteria;o__Myxococcales;f__Cystobacteraceae;g__Angiococcus	-0.56	4.1E-04
k_{COS}/k_{CO2}	16S rRNA	k__Bacteria;p__Proteobacteria;c__Alphaproteobacteria;o__Rhodospirillales;f__Acetobacteraceae;g__Roseomonas	-0.55	5.6E-04
k_{COS}/k_{CO2}	16S rRNA	k__Bacteria;p__Actinobacteria;c__Actinobacteria;o__Micrococcales;f__Micrococcaceae;g__Arthrobacter	-0.53	1.2E-03
k_{COS}/k_{CO2}	16S rRNA	k__Bacteria;p__Chloroflexi;c__Thermomicrobia;o__AKYG1722;Other;Other	-0.53	1.4E-03
k_{COS}/k_{CO2}	16S rRNA	k__Bacteria;p__Actinobacteria;c__Actinobacteria;o__Micrococcales;f__Promicromonosporaceae;g__Cellulosimicrobium	-0.53	1.5E-03
k_{COS}/k_{CO2}	16S rRNA	k__Bacteria;p__Gemmatimonadetes;c__Gemmatimonadetes;o__BD2_11_terrestrial_group;f__Unknown_Family_1002387;Other	-0.52	1.6E-03

k_{COS}/k_{CO2}	16S rRNA	k__Bacteria;p__Planctomycetes;c__Phycisphaerae;o__mle1_8;f__Unknown_Family_1002504;Other	-0.52	1.6E-03
k_{COS}/k_{CO2}	16S rRNA	k__Bacteria;p__Firmicutes;c__Clostridia;o__Clostridiales;Other;Other	-0.50	3.1E-03
k_{COS}/k_{CO2}	16S rRNA	k__Bacteria;p__Proteobacteria;c__Alphaproteobacteria;o__Sphingomonadales;f__Ellin6055;g__Unknown_Genus_1000756	-0.50	3.1E-03
k_{COS}/k_{CO2}	ITS2	p__Ascomycota;c__Leotiomycetes;o__Helotiales;f__Dermateaceae;g__Cryptosporiopsis;Other	0.70	5.8E-07
k_{COS}/k_{CO2}	ITS2	p__Ascomycota;c__Leotiomycetes;o__Helotiales;f__Dermateaceae;g__Unknown_22514;Other	0.66	8.5E-06
k_{COS}/k_{CO2}	ITS2	p__Ascomycota;c__Sordariomycetes;o__Hypocreales;f__Clavicipitaceae;g__Pochonia;Other	0.65	1.1E-05
k_{COS}/k_{CO2}	ITS2	p__Ascomycota;c__Leotiomycetes;o__Leotiales;f__Leotiaceae;Other;Other	0.64	1.1E-05
k_{COS}/k_{CO2}	ITS2	p__Zygomycota;c__Unknown_27287;o__Mucorales;f__Cunninghamellaceae;g__Absidia;Other	0.64	1.1E-05
k_{COS}/k_{CO2}	ITS2	p__Ascomycota;c__Leotiomycetes;o__Unknown_22440;f__Unknown_22441;Other;Other	0.64	1.1E-05
k_{COS}/k_{CO2}	ITS2	p__Basidiomycota;c__Microbotryomycetes;Other;Other;Other;Other	0.64	1.2E-05
k_{COS}/k_{CO2}	ITS2	p__Ascomycota;c__Leotiomycetes;o__Helotiales;Other;Other;Other	0.63	1.2E-05
k_{COS}/k_{CO2}	ITS2	p__Ascomycota;c__Dothideomycetes;o__Venturiales;f__Venturiaceae;Other;Other	0.62	2.1E-05
k_{COS}/k_{CO2}	ITS2	p__Ascomycota;c__Leotiomycetes;o__Helotiales;f__Hyaloscyphaceae;Other;Other	0.61	5.1E-05
k_{COS}/k_{CO2}	ITS2	p__Ascomycota;c__Leotiomycetes;o__Helotiales;f__Unknown_22483;g__Leptodontidium;Other	0.60	6.1E-05
k_{COS}/k_{CO2}	ITS2	p__Ascomycota;c__Unknown_21606;Other;Other;Other;Other	0.60	6.1E-05
k_{COS}/k_{CO2}	ITS2	p__Ascomycota;c__Leotiomycetes;o__Unknown_22453;f__Myxotrichaceae;g__Oidiodendron;Other	0.59	8.9E-05
k_{COS}/k_{CO2}	ITS2	p__Ascomycota;c__Leotiomycetes;o__Unknown_22453;f__Myxotrichaceae;Other;Other	0.59	9.2E-05
k_{COS}/k_{CO2}	ITS2	p__Ascomycota;c__Leotiomycetes;o__Unknown_22440;Other;Other;Other	0.58	1.2E-04
k_{COS}/k_{CO2}	ITS2	p__Zygomycota;c__Unknown_27299;o__Mucorales;f__Umbelopsidaceae;g__Umbelopsis;Other	0.58	1.4E-04
k_{COS}/k_{CO2}	ITS2	p__Ascomycota;c__Leotiomycetes;o__Helotiales;f__Dermateaceae;Other;Other	0.58	1.5E-04
k_{COS}/k_{CO2}	ITS2	p__Basidiomycota;c__Agaricomycetes;o__Agaricales;f__Physalacriaceae;g__Armillaria;Other	0.58	1.6E-04
k_{COS}/k_{CO2}	ITS2	p__Ascomycota;c__Leotiomycetes;o__Helotiales;f__Vibrisseaceae;Other;Other	0.57	1.9E-04
k_{COS}/k_{CO2}	ITS2	p__Ascomycota;c__Dothideomycetes;o__Mytilinidiales;Other;Other;Other	0.57	2.0E-04
k_{COS}/k_{CO2}	ITS2	p__Basidiomycota;c__Agaricomycetes;o__Agaricales;f__Cortinariaceae;g__Cortinarius;Other	0.57	2.2E-04

k_{COS}/k_{CO2}	ITS2	p__Ascomycota;c__Leotiomycetes;o__Unknown_22440;f__Unknown_22441;g__Meliniomyces;Other	0.57	2.3E-04
k_{COS}/k_{CO2}	ITS2	p__Ascomycota;c__Eurotiomycetes;o__Chaetothyriales;f__Herpotrichiellaceae;g__Capronia;Other	0.56	2.5E-04
k_{COS}/k_{CO2}	ITS2	p__Ascomycota;c__Eurotiomycetes;o__Chaetothyriales;f__Herpotrichiellaceae;g__Unknown_22134;Other	0.56	2.8E-04
k_{COS}/k_{CO2}	ITS2	p__Cercozoa;c__Unknown_27606;o__Unknown_27607;Other;Other;Other	0.56	2.8E-04
k_{COS}/k_{CO2}	ITS2	p__Ascomycota;c__Leotiomycetes;o__Unknown_22453;f__Myxotrichaceae;g__Gymnostellatospora;Other	0.56	3.4E-04
k_{COS}/k_{CO2}	ITS2	p__Ascomycota;c__Sordariomycetes;o__Hypocreales;f__Cordycipitaceae;Other;Other	0.55	3.8E-04
k_{COS}/k_{CO2}	ITS2	p__Basidiomycota;c__Agaricomycetes;o__Agaricales;f__Inocybaceae;g__Inocybe;Other	0.55	4.7E-04
k_{COS}/k_{CO2}	ITS2	p__Basidiomycota;c__Agaricomycetes;o__Thelephorales;f__Thelephoraceae;g__Tomentella;Other	0.54	5.2E-04
k_{COS}/k_{CO2}	ITS2	p__Basidiomycota;c__Agaricomycetes;o__Agaricales;f__Mycenaceae;g__Mycena;Other	0.54	5.5E-04
k_{COS}/k_{CO2}	ITS2	p__Ascomycota;c__Leotiomycetes;o__Helotiales;f__Rutstroemiaceae;Other;Other	0.54	6.0E-04
k_{COS}/k_{CO2}	ITS2	p__Basidiomycota;c__Agaricomycetes;o__Agaricales;f__Strophariaceae;g__Pholiota;Other	0.54	6.2E-04
k_{COS}/k_{CO2}	ITS2	p__Ascomycota;c__Leotiomycetes;o__Unknown_22438;f__Unknown_22439;g__Leohumicola;Other	0.54	6.6E-04
k_{COS}/k_{CO2}	ITS2	p__Basidiomycota;c__Microbotryomycetes;o__Unknown_26508;f__Unknown_26509;Other;Other	0.54	6.6E-04
k_{COS}/k_{CO2}	ITS2	p__Ascomycota;c__Sordariomycetes;o__Coniochaetales;f__Coniochaetaceae;Other;Other	0.53	6.6E-04
k_{COS}/k_{CO2}	ITS2	p__Ascomycota;c__Eurotiomycetes;o__Chaetothyriales;f__Herpotrichiellaceae;g__Unknown_22066;Other	0.53	9.5E-04
k_{COS}/k_{CO2}	ITS2	p__Ascomycota;c__Eurotiomycetes;o__Chaetothyriales;f__Herpotrichiellaceae;g__Unknown_22137;Other	0.52	1.1E-03
k_{COS}/k_{CO2}	ITS2	p__Zygomycota;c__Unknown_27299;o__Mucorales;f__Mucoraceae;Other;Other	0.52	1.2E-03
k_{COS}/k_{CO2}	ITS2	p__Ascomycota;c__Dothideomycetes;o__Hysteriales;f__Gloniaceae;g__Cenococcum;Other	0.51	1.4E-03
k_{COS}/k_{CO2}	ITS2	p__Ascomycota;c__Unknown_21650;o__Unknown_21651;f__Pseudeurotiaceae;g__Unknown_21652;Other	0.51	1.6E-03
k_{COS}/k_{CO2}	ITS2	p__Ascomycota;c__Leotiomycetes;o__Unknown_22426;f__Unknown_22427;g__Collophora;Other	0.51	1.6E-03
k_{COS}/k_{CO2}	ITS2	p__Ascomycota;c__Sordariomycetes;o__Xylariales;f__Xylariaceae;g__Nemania;Other	0.50	2.3E-03
k_{COS}/k_{CO2}	ITS2	p__Chlorophyta;Other;Other;Other;Other;Other	-0.63	1.2E-05
k_{COS}/k_{CO2}	ITS2	p__Ascomycota;c__Sordariomycetes;o__Sordariales;f__Chaetomiaceae;Other;Other	-0.60	5.8E-05
k_{COS}/k_{CO2}	ITS2	p__Ascomycota;c__Sordariomycetes;o__Sordariales;f__Chaetomiaceae;g__Chaetomium;Other	-0.58	1.6E-04

k_{COS} /k_{CO2}	ITS2	p__Ascomycota;c__Sordariomycetes;o__Hypocreales;f__Unknown_23151;g__Acremonium;Other	-0.55	3.4E-04
k_{COS} /k_{CO2}	ITS2	p__Chytridiomycota;c__Chytridiomycetes;o__Rhizophlyctidales;f__Rhizophlyctidaceae;Other;Other	-0.53	6.8E-04
k_{COS} /k_{CO2}	ITS2	p__Cercozoa;c__Unknown_27634;Other;Other;Other;Other	-0.51	1.6E-03
k_{COS} /k_{CO2}	ITS2	p__Chlorophyta;c__Unknown_27544;o__Unknown_27545;f__Unknown_27546;Other;Other	-0.50	2.0E-03
k_{COS} /k_{CO2}	ITS2	p__Chlorophyta;c__Unknown_27544;o__Unknown_27545;f__Unknown_27546;g__Unknown_27547;Other	-0.50	2.0E-03

Table S8 Spearman correlations of catalyzed reaction rates for COS (k_{COS}), CO_2 (k_{CO2}), and their ratio (k_{COS}/k_{CO2}) with phylum-level OTU taxonomic ranks (Level 2) for OTUs with correlation coefficients > |0.5| and adjusted p-values < 0.01. Significant correlations indicated in yellow when positive and in in orange when negative. Data in this table are listed first by k_{CA} type (k_{COS}, k_{CO2}, k_{COS}/k_{CO2}), then by amplicon target (16S rRNA followed by ITS2), and finally sorted by correlation strength (positive correlations followed by absolute strength of negative correlations).

k_{CA}	**Amplicon**	**Taxon**	**Correlation**	**Adjusted p-value**
k_{COS}	16S rRNA	k__Bacteria;p__Nitrospirae	-0.51	1.0E-03
k_{COS}	ITS2	p__Cercozoa;c__Unknown_27586	0.54	8.5E-04
k_{COS}	ITS2	p__Chlorophyta;Other	-0.51	2.4E-03
k_{COS}	ITS2	p__Cercozoa;c__Unknown_27634	-0.55	8.5E-04
k_{CO2}	16S rRNA	na	na	na
k_{CO2}	ITS2	na	na	na
k_{COS} /k_{CO2}	16S rRNA	k__Bacteria;p__Acidobacteria	0.69	3.6E-08
k_{COS} /k_{CO2}	16S rRNA	k__Bacteria;p__Nitrospirae	-0.65	4.3E-07
k_{COS} /k_{CO2}	16S rRNA	k__Bacteria;p__Chloroflexi	-0.54	1.4E-04
k_{COS} /k_{CO2}	16S rRNA	k__Bacteria;p__Deinococcus_Thermus	-0.51	4.0E-04
k_{COS} /k_{CO2}	ITS2	p__Ascomycota;c__Unknown_21339	0.56	2.4E-04
k_{COS} /k_{CO2}	ITS2	p__Ascomycota;c__Unknown_21650	0.56	2.4E-04
k_{COS} /k_{CO2}	ITS2	p__Ascomycota;c__Unknown_21606	0.53	6.5E-04
k_{COS} /k_{CO2}	ITS2	p__Cercozoa;c__Unknown_27586	0.52	8.2E-04
k_{COS} /k_{CO2}	ITS2	p__Cercozoa;c__Unknown_27634	-0.64	5.7E-06

k_{COS}/k_{CO2}	ITS2	p__Chlorophyta;Other	-0.63	5.7E-06
k_{COS}/k_{CO2}	ITS2	p__Basidiomycota;c__Ustilaginomycetes	-0.53	5.9E-04
k_{COS}/k_{CO2}	ITS2	p__Chlorophyta;c__Unknown_27544	-0.51	1.1E-03

Table S9 Soil metatranscriptome size and CA recovery statistics assembled CA and CA reads for the CA classes observed in soil. Table sorted by metatranscriptome size.

				**Assembled CA**				**CA reads (gene copies)**			
**Site Name**	**IMG Submission ID**	**JGI Project ID**	**Genome Size (bp)**	**Total**	**α**	**β**	**γ**	**Total**	**α**	**β**	**γ**
Carnegie Cornfield, CA	101514	1106757	681529179	301	4	280	17	763	7	635	121
Willow Creek, WI	92749	1106758	671257491	401	8	367	26	916	16	730	170
Great Plains, OK	92758	1106759	587647789	403	0	358	45	1157	0	839	318
Stunt Ranch 2, CA	101513	1106756	521748598	183	6	163	14	410	12	322	76
Big Basin, CA	92728	1106760	303323377	142	3	134	5	328	6	307	15
Jasper Ridge SND, CA	92730	1106761	245908234	97	0	94	3	216	0	210	6
Kohala Peninsula, HI	92729	1106762	244931251	141	2	136	3	255	4	223	28
Dry Field, Cambodia	92731	1106763	146941622	59	1	58	0	132	2	130	0
Stunt Ranch 1, CA	92732	1106764	88712635	13	0	13	0	20	0	20	0
Bondville, IL	92727	1106755	65589854	12	0	11	1	40	0	27	13

Supplemental Figures

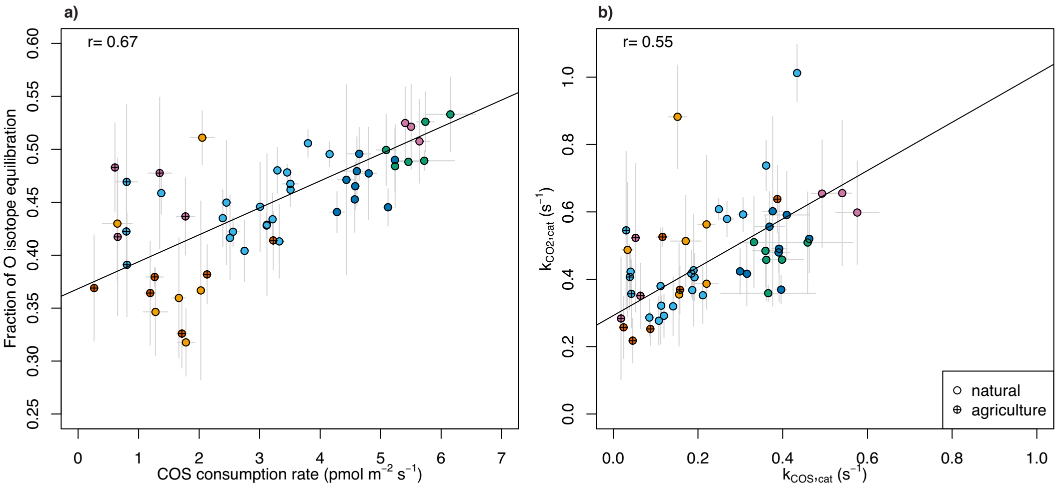

Figure S1 Correlations between soil **a)** COS consumption and the degree of equilibration (f_{eq}) of oxygen isotopes between incoming CO_2 and soil water ($r = 0.67$, $p < 0.001$) and **b)** enzyme catalyzed first order reaction rates for COS (k_{COS}) and CO_2 (k_{CO2}) ($r = 0.55$, $p < 0.001$) (peat samples excluded). Biome indicated by color key as in Fig. 1 and agricultural sites denoted by ⊕ symbol.

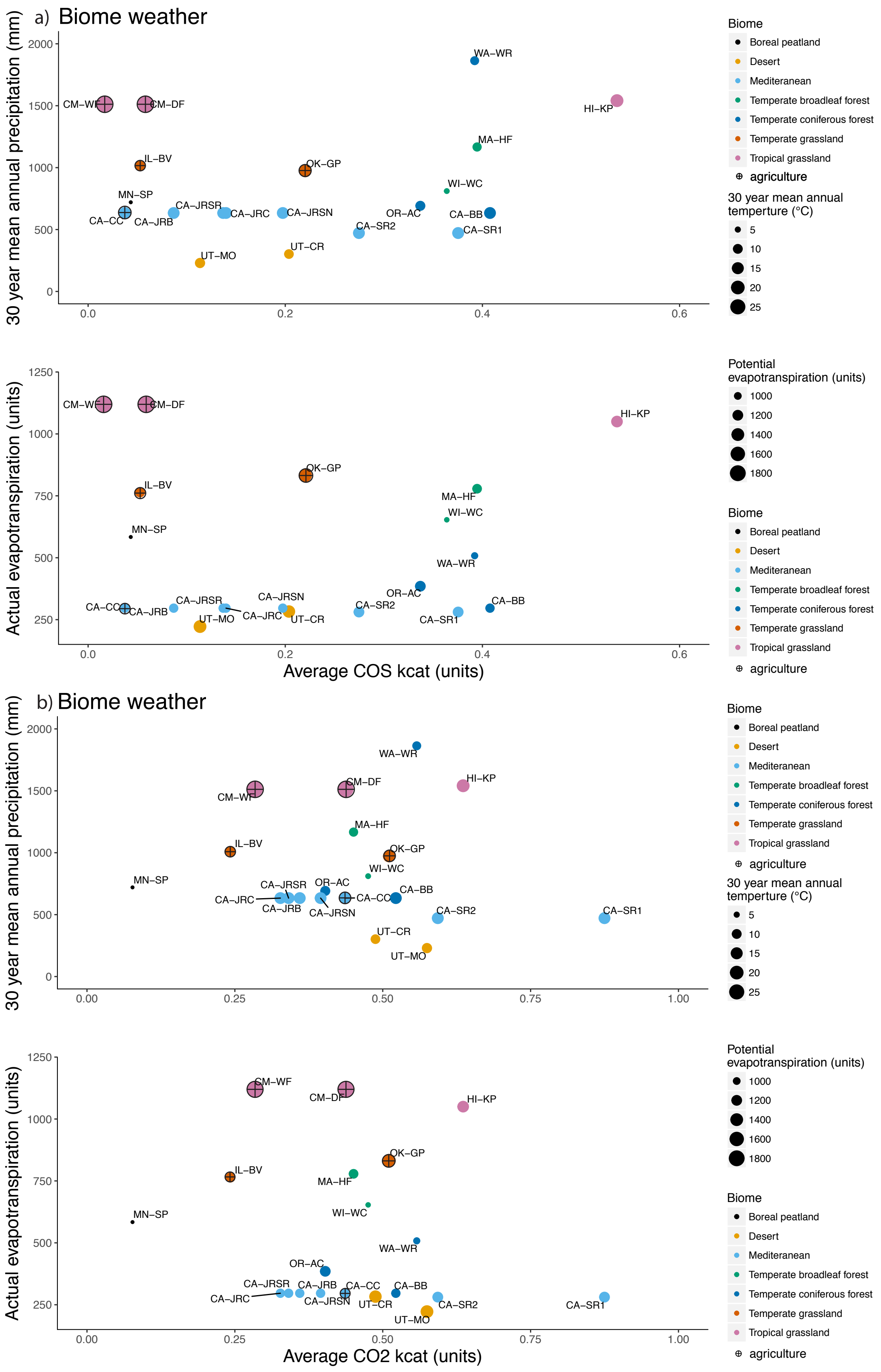
a)
Biome weather
30 year mean annual precipitation (mm)
Actual evapotranspiration (units)
Average COS kcat (units)
b)
Biome weather
30 year mean annual precipitation (mm)
Actual evapotranspiration (units)
Average CO2 kcat (units)
CM–WF
CM–DF
HI–KP
WA–WR
MA–HF
WI–WC
IL–BV
OK–GP
MN–SP
CA–CC
CA–JRB
CA–JRSR
CA–JRC
CA–JRSN
OR–AC
CA–BB
CA–SR2
CA–SR1
UT–MO
UT–CR
Biome
Boreal peatland
Desert
Mediterranean
Temperate broadleaf forest
Temperate coniferous forest
Temperate grassland
Tropical grassland
agriculture
30 year mean annual temperture (°C)
5
10
15
20
25
Potential evapotranspiration (units)
1000
1200
1400
1600
1800

Figure S2 Evaluation of biome, land use, and climate variables as descriptors for site conditions and relationship with CA-catalyzed reaction rates for COS and CO_2 in soil. Climate variables represented with y-axis and point size for mean annual precipitation (MAP) and mean annual temperature (MAT) in the top panels and actual evapotranspiration (AET) and potential evapotranspiration (PET) in the lower panels, respectively, versus site-average CA activity for **a)** COS and **b)** CO_2. Biome indicated by color key and agricultural sites denoted by ⊕ symbol. No single climate variable predicted trends in CA activity. For example, CA activity for COS in agricultural sites did not follow trends observed in agricultural sites (e.g., in **a**) increasing k_{COS} with MAP or AET). We use biome and land use to group sites to encapsulate climate variables and incorporate variability of soil properties and vegetation type for the purpose of describing differences between sites. Climatic data derived from 30-year averages (1984-2014) Climate Research Unit (CRU TS v3.23) data (Supplemental Methods).

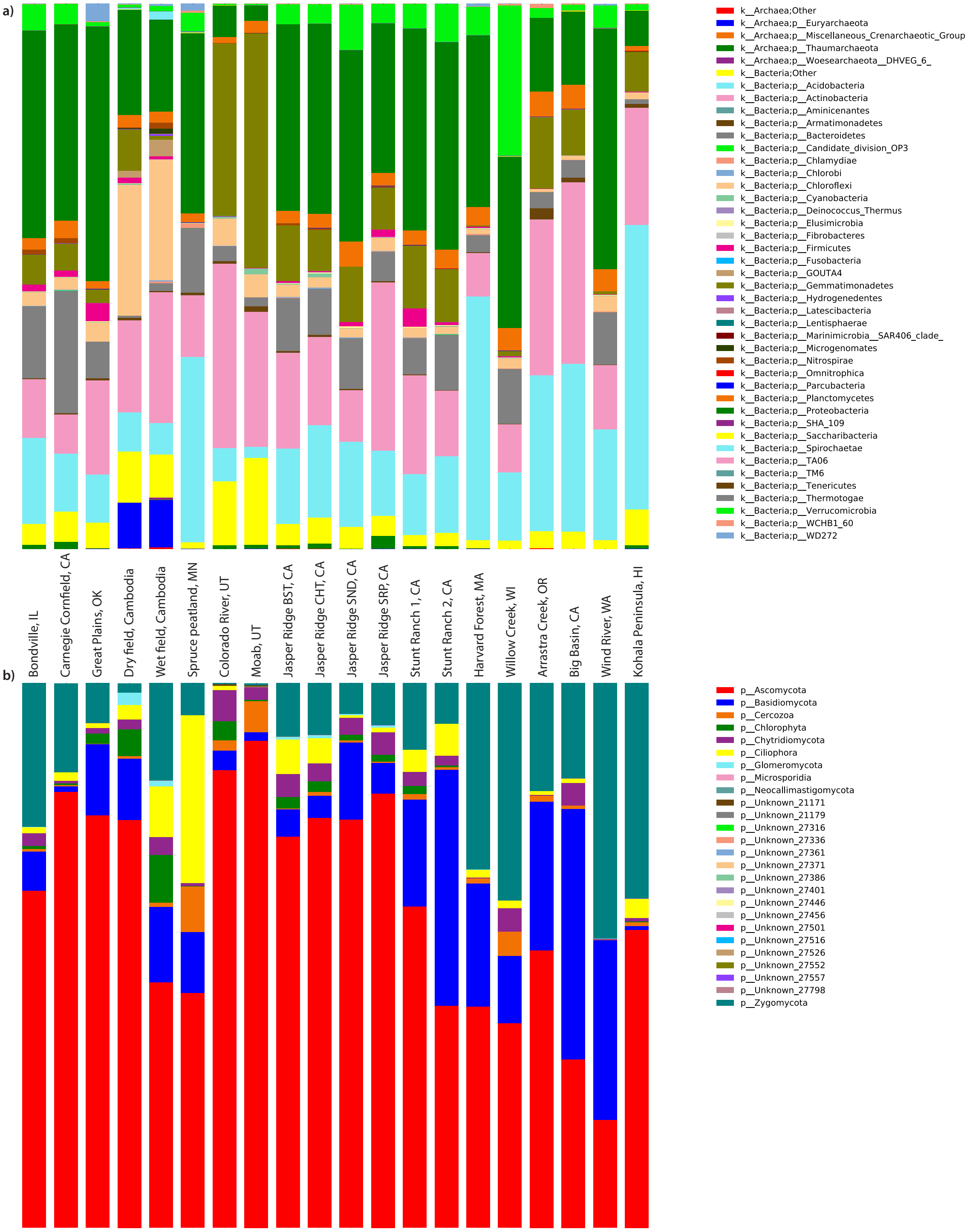

Figure S3 Phylum-level relative abundance **a)** Bacteria and Archaea and **b)** Microeukaryotes based on 16S rRNA and ITS2 phylogenetic gene amplicon sequences, respectively. Each bar represents the median of 3 replicates.

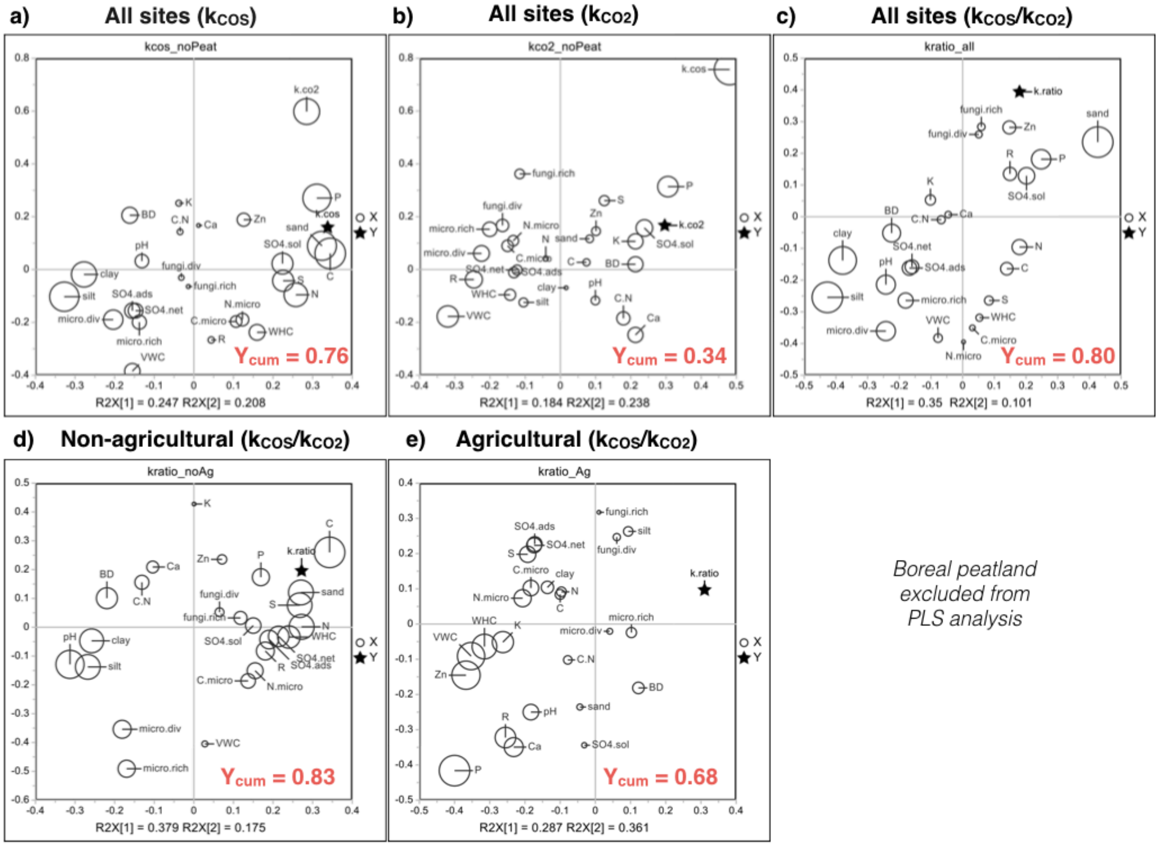

Figure S4 Partial least squares model of 25 edaphic factors explaining variance (total in red text) in k_{COS}, k_{CO2}, and their ratio of k_{COS}/k_{CO2} (denoted here as *k.cat.ratio*). Point size reflects the importance of parameters to describing variance in all soil properties. Factors correlated with the "Y" variable (black star) would fall on a line through the origin in the same quadrant (anti-correlated variables in the opposite quadrant). Additional results for the COS consumption rate ($F_{COS,consumption}$), the fractional equilibration of $CO^{18}O$ (f_{eq},), and COS deposition velocity ($V_{d,COS}$). Gas fluxes ($F_{COS,consumption}$ and f_{eq}) and CA-catalyzed reaction rates (k_{COS} and k_{CO2}) were significant reciprocal predictors (top predictor for at least one PLS component). Additional predictors of CA activity included nutrients, such as carbon (C), nitrogen (N), phosphorus (P) and sulfur (S), physical properties affecting gas diffusion in soils (texture, bulk density), Zn, a common metal cofactor in CA.

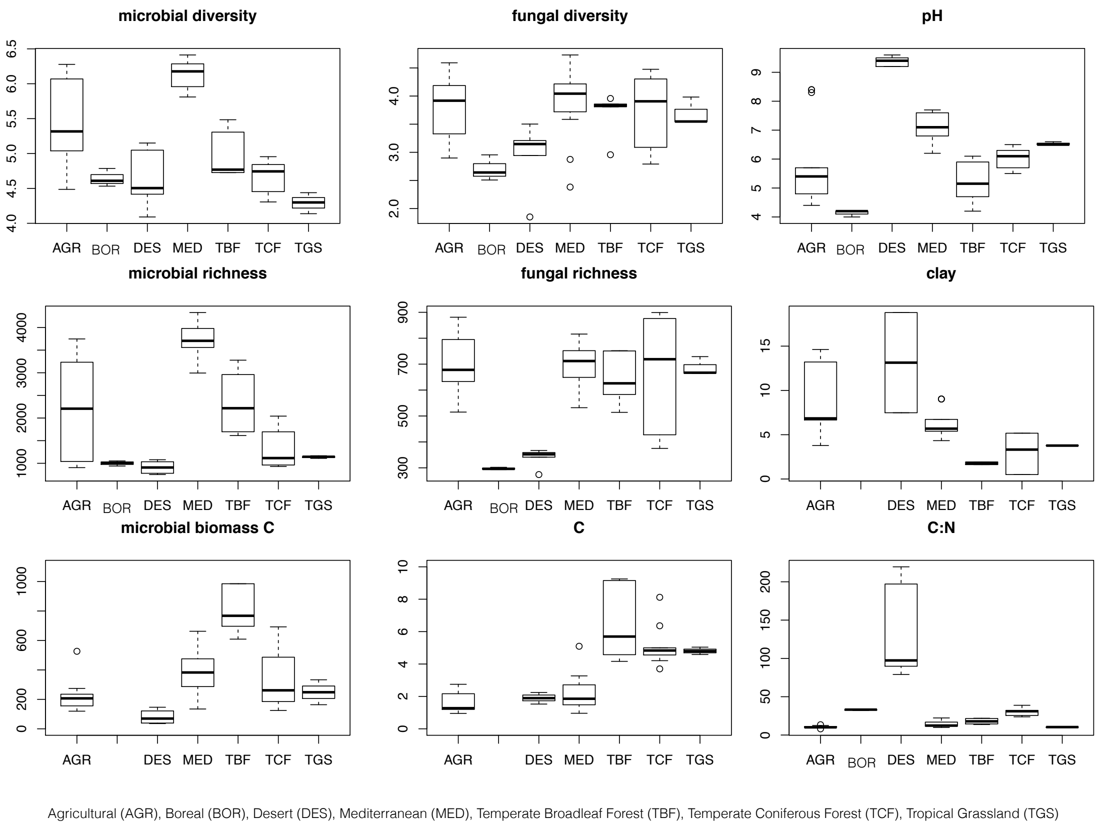

Figure S5 Biome-level boxplots of select soil chemical, physical, and microbial properties. Properties are unitless except for clay (%), microbial biomass C (μg C kg dry soil^{-1}), and C (%). Peat (BOR; boreal) soils not shown in cases where mass-weighting by dry weight leads to dramatically different values from other biomes (those data in Tables S3-S5).

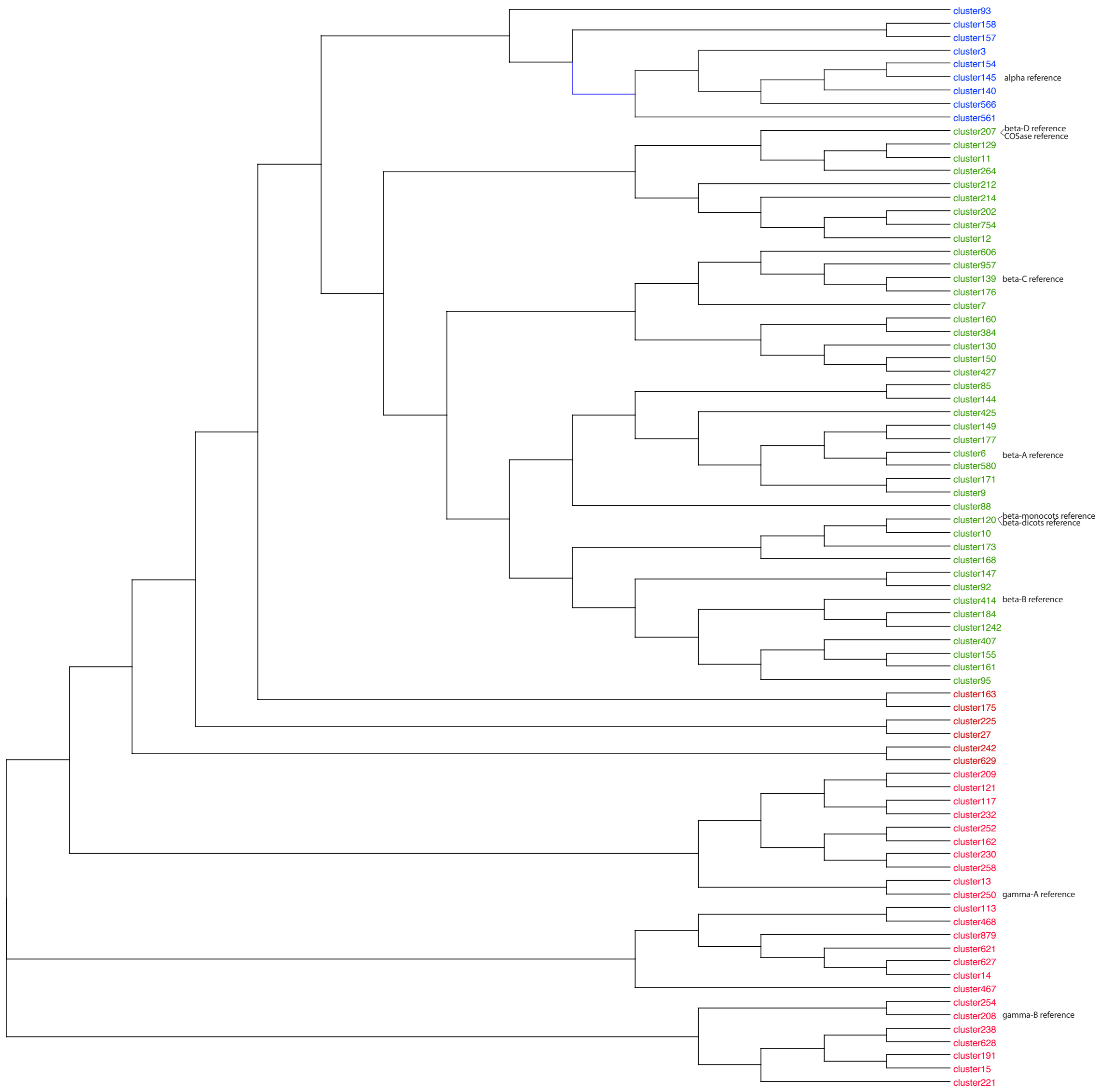

Figure S6 Clustal Omega tree of CA centroids from the 81 clusters with >50 CA. HMMs constructed from each cluster were run on CA of known class and clade (black text) ued to confirm CA classes and identify β-CA clades. Alpha, beta, and gamma CA listed in blue, green, and red, respectively.

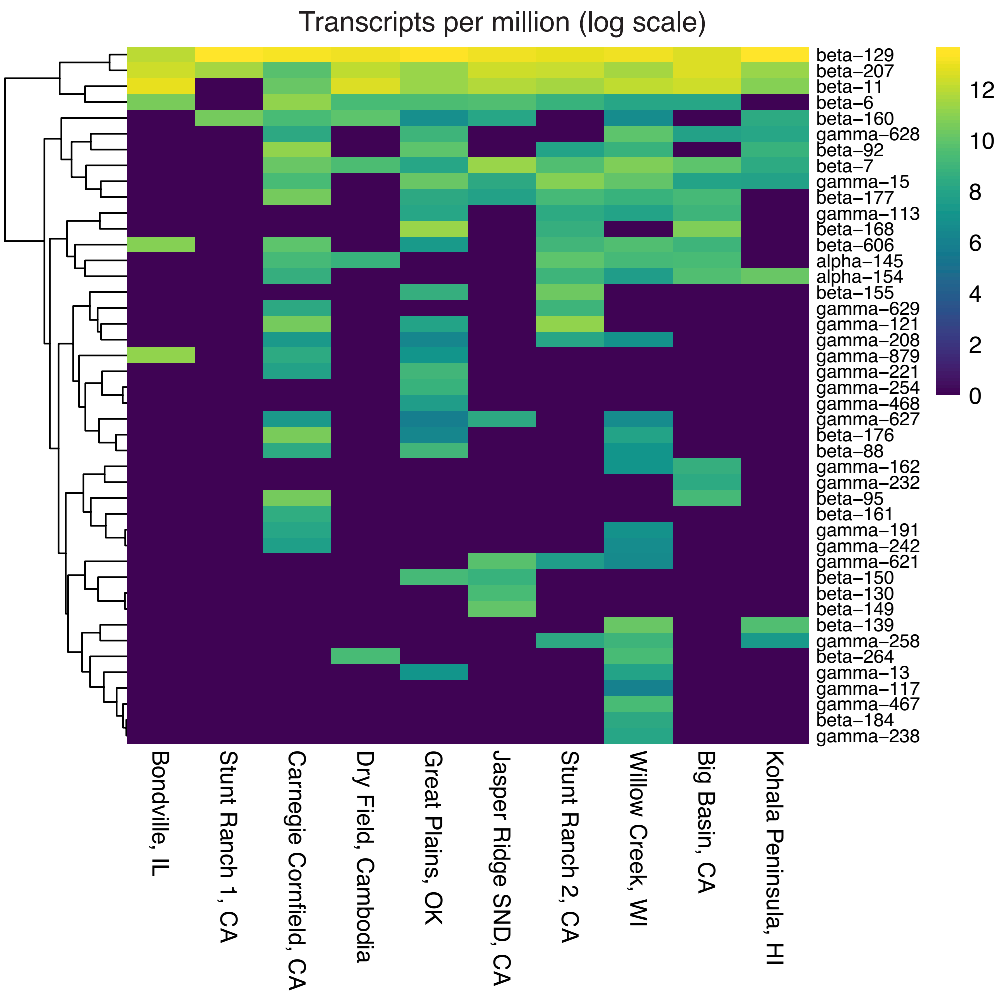

Figure S7 Patterns of CA gene expression in seven soil metatranscriptomes. Transcripts per million (log scale) recovered using the 81 CA HMMs shown for all clusters recovering at least one assembled CA in a given cluster across all sites. The highest levels of expression were observed for β-CA, though gene expression was not observed in all β-CA or other CA classes. Figure created using pheatmap R-package (Raivo Kolde). Row clustered with Euclidean distance matrix.

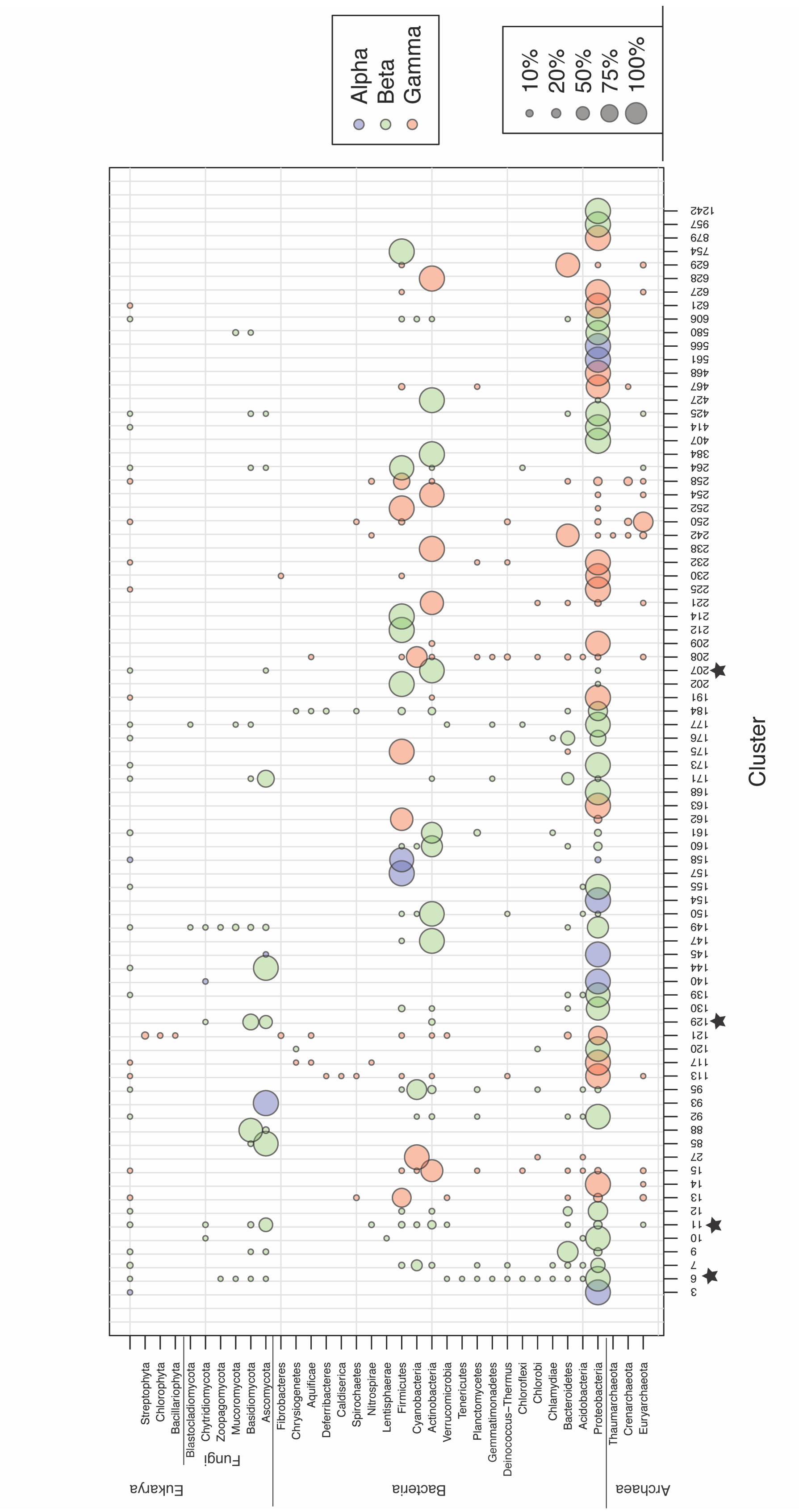

Alpha
Beta
Gamma
10%
20%
50%
75%
100%
Cluster
Eukarya
Fungi
Bacteria
Archaea
Streptophyta
Chlorophyta
Bacillariophyta
Blastocladiomycota
Chytridiomycota
Zoopagomycota
Mucoromycota
Basidiomycota
Ascomycota
Fibrobacteres
Chrysiogenetes
Aquificae
Deferribacteres
Caldiserica
Spirochaetes
Nitrospirae
Lentisphaerae
Firmicutes
Cyanobacteria
Actinobacteria
Verrucomicrobia
Tenericutes
Planctomycetes
Gemmatimonadetes
Deinococcus−Thermus
Chloroflexi
Chlorobi
Chlamydiae
Bacteroidetes
Acidobacteria
Proteobacteria
Thaumarchaeota
Crenarchaeota
Euryarchaeota
3
6
7
9
10
11
12
13
14
15
27
85
88
92
93
95
113
117
120
121
129
130
139
140
144
145
147
149
150
154
155
157
158
160
161
162
163
168
171
173
175
176
177
184
191
202
207
208
209
212
214
221
225
230
232
238
242
250
252
254
258
264
384
407
414
425
427
467
468
561
566
580
606
621
627
628
629
754
879
957
1242

Figure S8 Taxonomic composition associated with CA clusters. Shown are the phylum-level taxonomic identity CA genes with best hit to HMM for each cluster (lowest E-value). Color denotes CA class and point size the relative abundance of the associated phylum to the taxonomic composition within that cluster. The three most highly expressed clades in soil (Fig. S8) are denoted with black stars.
