# Supplementary material for: Soil exchange rates of COS and CO18O differ with the diversity of microbial communities and their carbonic anhydrase enzymes
Source: ISME J. 2018 Sep 13;13(2):290–300. doi: 10.1038/s41396-018-0270-2 (PMC6330096; doi:10.1038/s41396-018-0270-2)
Supplement: Supplementary file 2 — Supplemental Information [file 41396_2018_270_MOESM2_ESM.pdf]

Supplemental Information

S1: Methods for analysis of soil structure, chemistry, and microbial biomass

Physical soil properties. Gravimetric (g H_2O g^{-1} soil) soil water holding capacity (WHC) was determined from the mass difference before and after 48 h of 105°C drying of soils saturated (20 g soil in 40 mL nanopure water) for 2 hours in stoppered filter funnels (Whatman No. 42, GE Healthcare, Little Chalfont, UK) and then drained for 6 h [1]. Bulk density (BD) was determined from soil mass (approximately 10 g) and volume (measured in 15 mL conical tubes) for sieved soil used for gas exchange measurements. We report soil moisture as the volumetric water content (VWC; v/v) from the mass difference before and after 48 h of 105°C drying (volume conversion with BD). Soil texture was assessed using a multi-wavelength laser diffraction particle analyzer (LS 13 320 MW, Beckman Coulter, Brea, CA, USA) by Fraunhofer diffraction theory, which we converted for comparability with data derived using sedimentation methods [2].

Chemical soil properties. Soil pH was determined in 1:2.5 soil:water slurries hand-shaken and allowed to equilibrate for 2 hours before measurement. We used an elemental analyzer (NA-1500, Carlo-Erba, Milan, Italy) to measure soil C and N, and additional elements were measured by X-ray diffraction spectrometry (Xepos HE XRF Spectrometer, Spectro Analytical Instruments GmbH, Kleve, Germany). Sulfate was measured in moist and dry soils by ion chromatography (DX-500, Dionex, Thermo Scientific, Waltham, MA, USA) following potassium dihydrogen phosphate (0.016 M KH_2PO_4) or deionized water extractions (1:5 soil to solution, 2 hours shaking, centrifuged, filtered 0.2 μm) for total and soluble inorganic sulfate, respectively, thus revealing adsorbed sulfate by difference.

Microbial biomass. Soils for microbial biomass determination were collected following the moist soil gas exchange rates and were stored in the refrigerator until analysis. Microbial biomass was determined by chloroform fumigation of 3 g of soil dry weight equivalent ($g_{soil,dw}$) for three days in the dark following S. E. Hobbie, (1998) (http://web.stanford.edu/group/Vitousek/cfde.htm). Fumigated and non-fumigated control soils were extracted with 10 mL 0.5 K_2SO_4 through pre-leached (using 0.5 M K_2SO_4) filter paper (Whatman No. 1, GE Healthcare, Little Chalfont, UK) after one hour of vigorous shaking. Extracts were frozen until dilution (1:3 into DI water) and analyzed on a total organic carbon analyzer (TOC-L, Shimadzu, Tokyo, Japan). The chloroform-labile pool of C or N (EC and EN; ug C or ug N $g_{soil,dw}^{-1}$) was estimated from the difference between the extracted C or N in fumigated and control samples and converted to microbial biomass (C or N) using $C=EC/k_{EC}$ or $N=EN/k_{EN}$, where k_{EC} and k_{EN} representing the C and N mineralization efficiency, taken here to be the constants 0.45 and 0.54, respectively [3, 4].

Site climate data: Climate data for each sampling site was reported as the 30-year mean (1984-2014) of mean annual precipitation (MAP), temperature (MAT), actual evapotranspiration (AET), and potential evapotranspiration (PET) from the Climate Research Unit (CRU TS v3.23) time series data at a 0.5°x0.5° resolution containing the site (or nearest grid point above the land-fraction threshold for the Kohala Peninsula, Hawaii site) [5].

S2: Extended methods for soil, soil incubations, and soil subsampling

Initial soil processing and storage. Replicates from each site were not homogenized: replicates were collected, sieved (2.00 mm; Humboldt Mfg. Co., Elgin, IL,

USA), and refrigerated separately. The fraction of sieved soils to litter and rocks was recorded. In the median, soils were sieved within a median of 9 days of sampling and stored in the refrigerator for a median of 46 days until incubation.

Soil pre-incubations. Before gas exchange measurements, soils were air dried for a median of 3 days (typically to 6% H_2O by weight) in sterile 25 cm^2 petri dishes partially covered by lids to remove excess soil moisture. Duplicate sets of the 3 replicates from each site were pre-incubated for exactly 7 days at 30% WHC. For each replicate, two sets of 80 g dry soil equivalent were transferred to sterilized half pint mason jars and soil moisture was adjusted to 30% WHC with sterile nanopure water. The mason jars were covered with parafilm (sterilized in a 3% H_2O_2 bath) with a cotton ball inserted into a 1 cm diameter puncture hole to allow headspace gas exchange during incubation. Mason jars were placed in approximately 40 L plastic environmental chambers containing a few cm of water (same water used to adjust soil moisture) to modulate soil temperature and mitigate the air-soil moisture deficit. Environmental chambers were incubated within a larger insulated chamber at room temperature (around 22.5°C) for 7 days in the dark. The wetted samples were transferred after 6 days to 1 L PFA chambers (100-1000-01, Savillex, Eden Prairie, MN, USA) and covered with sterilized parafilm to settle for the remaining 24 hours until gas exchange measurements for the moist soil set. After 7 days of pre-incubation, the set of soils dry gas exchange measurements were transferred to aluminum trays to air dry for a median of 45 days. Moist peat samples were pre-incubated a second time following a 38-day frozen period. Following the dynamic soil gas exchange measurement, moist soils were destructively sampled for soil moisture, pH, sulfate, microbial biomass determination, and DNA/RNA extractions.

S3: Extended methods for COS exchange measurements

COS exchange measurements at 30% WHC. Following pre-incubation, "wet" soils in PFA chambers were installed on a dynamic flow-through chamber soil flux system for COS using the same experimental setup as presented by Whelan *et al.*, (2016). Briefly, COS, CO_2, and H_2O mole fractions were measured using a quantum cascade laser (QCL, Aerodyne Research, Inc., Billerica, MA, US) in air flowing at approximately 0.3 L min^{-1} either through the soil chamber or a chamber-bypass line representing concentrations at the *outlet* and *inlet* of the soil chamber, respectively. The inlet air was humidified using nafion tubing placed in water and soil chambers were placed in a water bath for temperature control. Inlet air flowed into the PFA soil chambers (14 cm height) from an inlet in top of the chamber lid and out from an outlet line (1/8" PTFE) extending into the center of the jar (8 cm depth from the lid). We modified the experimental setup of [6] to include an averaging volume (2 L PFA chamber) on the gas inlet line. Wetted soil measurements were made at 20°C and the inlet gas composition was set using a mass flow control system from which we added CO_2 from a pure tank to synthetic air scrubbed of CO_2 and H_2O with a soda lime and Drierite trap at the desired mole fraction of 450 parts per million (ppm; e^{-6}). A temperature- and pressure-controlled permeation tube was used to add COS to the controlled gas mixture at around 450 parts per trillion (ppt; e^{-12}). Mole fractions were measured during three cycles of the following 40-min program for each wet soil replicate: bypass flow (10 min; inlet), N_2 tank (10 min; zero), and soil chamber flow (20 min; outlet).

COS exchange measurements in dry soils. The dry soils were measured with a 1-hour program, bypass flow (10 min), N_2 tank (10 min), and soil chamber flow (40 min),

and over a temperature ramp from 10°C to 40°C. The same experimental setup was used, but inlet air was drawn from ambient laboratory air instead of the defined gas mixture described for wet soils and samples for isotopic analysis were not collected.

Data processing. Measurements were averaged at the end of each stage of the dynamic flow program (when mole fractions stabilize) over intervals of 4 min for N_2 and bypass and 8 min for chamber measurements. We corrected COS and CO_2 mole fraction measurements for water vapor dilution and spectral interferences [6, 7] using instrument-specific water vapor dependencies to adjust mole fractions to a common humidity level of 10 parts per thousand (ppth; e^{-3}) H_2O. Water vapor dependency: COS (ppb) = m*H_2O (ppb) + b, where m = -1.05e-09 and b= 0.539 and CO_2 (ppm) = M*H_2O (ppth) + B, where M = -0.734 and B= 466. Water vapor corrections were 3% (5%) and 2% (4%) in the median (95th percentile) for COS and CO_2, respectively. Instrument drift was corrected for by subtraction of baseline N_2 tank measurements interpolated onto bypass and chamber measurements. The QCL instrument (Aerodyne Research, Inc., Billerica, MA, US) precision is 2 ppt for COS, 0.03 ppm for CO_2, and 3 ppm for H_2O at 60 s averaging with an absolute calibration accuracy of 5% for COS and CO_2 [8].

COS net gas exchange calculations. The exchange rate (F) of COS and CO_2 were determined from the difference between mole fractions at the outlet (c_o) and inlet (c_i) measured, respectively, by the chamber and bypass flow. For example, for COS:

$$F_{\mathrm{COS}} = \frac{u}{S}\left(c_{\mathrm{o,COS}} - c_{\mathrm{i,COS}}\right) \quad (1)$$

where u (mol s^{-1}) is the flow rate of air through the chamber, S (0.0078 m^2) is the soil surface area and $c_{\mathrm{o,COS}}$ and $c_{\mathrm{i,COS}}$ are the dry air COS mixing ratios inside the chamber and the bypass air, respectively. The mixing ratios inside the chamber were also converted into volumetric concentration using air temperature measurements in the chamber and assuming an atmospheric pressure of 101.325 kPa.

COS gas exchange partitioning. COS emissions dry soils were measured at 20°C as described above. Following others [6] we assumed that COS emissions from air-dried soils well approximated the COS production rates at 30% soil WHC. This assumption is based on the idea that, upon drying, only the consumption of COS through CA-catalyzed hydrolysis is inhibited, which is coherent with [9] who observed little difference on the COS uptake rates at 0% and 30% WHC after inhibiting soil COS consumption with the fungicide nystatin and with a recent comparison of moist and dry soil production rates by [10]. Thus, we assumed that the COS net exchange in dry soils only represents COS production, which we used to partition the net COS exchange measured in wet soils at 20°C and determine COS consumption using $F_{\mathrm{COS}} = F_{\mathrm{COS,production}} + F_{\mathrm{COS,consumption}}$ with $F_{\mathrm{COS,production}} = F_{\mathrm{COS,dry}}$. Accounting for the source term improved the correlation between CA activity for COS and CO_2, but only by 5% and did not significantly impact the significance of the correlation, and the conclusions of this study have low sensitivity to uncertainty in the COS source term.

S4: Extended methods for $CO^{18}O$ exchange measurements

Oxygen isotopes of carbon dioxide. We report $^{18}O/^{16}O$ molar ratios of a sample (R_{sample}) in delta notations, as deviation (in per mil) from international standards ($R_{standard}$): $\delta_{sample} = \left(R_{sample}/R_{standard}\right) - 1$. The standard for water is Vienna Standard Mean

Ocean Water (VSMOW) and the standard for CO_2 is Vienna Pee Dee Belemnite (VPDB-CO_2).

$CO^{18}O$ exchange measurements. In addition to the previously mentioned experimental setup, we also added a sampling port, which was a stainless steel tee fitting with a septum (Thermogreen®, Supelco, Bellefonte, Pennsylvania, USA), on the common outlet stream from the chamber and bypass flow. Enriched water ($\delta^{18}O$-H_2O = 47.57±0.03 ‰ VSMOW) was used to adjust the soil moisture for the wet soils, and a pure CO_2 tank was used to supply a steady mole fraction (450 ppm) of ^{18}O-depleted CO_2 ($\delta^{18}O$-CO_2 of approximately -20‰ VPDB-CO_2) into the gas chamber inlet. Samples for flow-through CO_2 isotopic analysis were withdrawn during soil chamber sampling in the final minute of any given 20-minute soil chamber flow measurement. Two samples were withdrawn during the bypass flow sampling to bracket all of the exchange measurements for a given measurement set (i.e., during bypass stream sampling before and after each set of 9 measurements; 3 replicates per site measured 3 times each). To sample, we used a 25-mL gas-tight syringe to evacuate a glass vial (538W Exetainer® 12ml round bottom with white cap, Labco Limited, Ceredigion, UK) that had been previously flushed with N_2 for 2 min, withdrew approximately 25 mL of gas from the sampling port, and injected the sample into the vials to a slight over pressure. Directly after each wet dynamic exchange measurement, the soil not used for destructive sampling (approximately 60 $g_{soil,dw}$) was added to 950 cm^3 mason jars, which were sealed and sampled through a septum port to determine the CO_2 isotopic composition after 1, 2, and 3 days of equilibration at 20°C in a controlled environmental chamber.

CO_2 isotope measurement, calibration, and precision. Vials were shipped from Stanford, CA to Forschungszentrum Jülich, Germany for analysis and were measured within 2-4 months from the time of gas collection depending on the sample. Both $\delta^{18}O$-CO_2 and $\delta^{13}C$-CO_2 of our samples were analyzed with a continuous-flow isotope-ratio mass spectrometer (CF-IRMS, IsoPrime 100, Elementar Analysensysteme, Langenselbold, Germany) coupled with a TraceGas unit (Elementar Analysensysteme) for pre-concentration of sample gas. Values were calibrated against two reference gas working standards (Standard 1: 400 ppm CO_2 in helium, $\delta^{13}C$-CO_2: -36.52‰, $\delta^{18}O$-CO_2: -26.17‰ vs. VPDB-CO_2, Air Liquide, Germany; Standard 2: 400 ppm CO_2 in helium, $\delta^{13}C$-CO_2: -4.07‰, $\delta^{18}O$-CO_2: -14.51‰ vs. VPDB-CO_2, Linde AG, Germany) that were analyzed in the same way as the samples. Measurement precision as defined as the standard deviation of six replicate analyses of the same gas ranged between 0.05‰ and 0.20‰ for both $\delta^{18}O$-CO_2 and $\delta^{13}C$-CO_2. Three other reference gases were used for cross-checking of measurement accuracy at concentrations and isotope values within and out of the range spanned by the two working standards (Standard 3: 3000 ppm CO_2 in helium, $\delta^{13}C$-CO_2: -36.65‰, $\delta^{18}O$-CO_2: -24.69‰ vs. VPDB-CO_2, Air Liquide, Germany; Standard 4: 3000 ppm CO_2 in helium, $\delta^{13}C$-CO_2: -35.45‰, $\delta^{18}O$-CO_2: -32.51‰ vs. VPDB-CO_2, Air Liquide, Germany; Standard 5: dried ambient air with 394.32 ppm CO_2, $\delta^{13}C$-CO_2: -8.55‰, $\delta^{18}O$-CO_2 vs. VPDB-CO_2: -0.53‰, Max Planck Institute for Biogeochemistry, Jena, Germany). Analytical accuracy as defined as the difference between delta values after application of the calibration function to raw analysis results and the true delta value of the standard gases varied between 0.11…0.15‰ and 0.01…0.19‰ for $\delta^{13}C$-CO_2 and $\delta^{18}O$-CO_2, respectively, within the range of delta values spanned by the two working Standards 1 and 2, and between 0.28…0.50‰ and 0.06…0.59‰ for delta values out of the range of delta values

spanned by working standards 1 and 2. The Standards 1-4 had been calibrated against primary carbonate standards prior to the measurements with a CF-IRMS (Delta Plus XP, ThermoFisher, Dreieich, Germany) coupled to a Gasbench (ThermoFisher). Standard 5 had been calibrated by the Max Planck Institute against primary gas standards from NIST. The measurement precision of CO_2 isotopes was exceptionally high CO_2. However, we collected samples for isotope analysis from the gas stream or chamber headspace via syringe at discrete intervals during the soil flux measurements, and sample vials were shipped before analysis. We anticipate that these steps associated with discrete sampling may have propagated noise into the $CO^{18}O$ measurements and could be the source for higher uncertainty in $CO^{18}O$ than COS measurements.

$CO^{18}O$ exchange data processing and calculations. The fraction of CO_2 flowing through the chamber that fully equilibrates with soil water (f_{eq}) was determined from the isotopic composition of CO_2 measured at the dynamic chamber inlet and outlet (δ_i and δ_o, respectively) and inside the sealed equilibration chamber (δ_{eq}). We determined δ_i as the average of chamber inlet (bypass) measurements bracketing a set of given “wet” soil exchange measurements (3 measurements of 3 replicates over 6 hours). The equilibrium isotopic signature, δ_{eq}, was determined from measurements on the sealed equilibration jars averaged for day 2 and 3. Exceptions included the two desert samples (Colorado River and Moab, Utah) (measured on day 1 only) and Carnegie Cornfield, CA (day 2 and 3 measurements overloaded by high CO_2 concentrations).

Because CO_2 respired from the soils is likely already equilibrated with soil water by soil CA this may cause an overestimation in the fraction of inlet CO_2 that invaded the soil matrix and equilibrated O isotopes with soil water. We accounted for contributions of respired CO_2 to the chamber outlet $\delta^{18}O_o$ by assuming that the moles of $CO^{18}O$ on the outlet of the chamber, $C_o\delta_o$, was the sum of the respired $CO^{18}O$ moles, $\delta_{eq}(C_o - C_i)$, a mole fraction $f_{eq}C_i\delta_{eq}$ that had equilibrated with soil water and a remaining mole fraction $(1 - f_{eq})C_i\delta_i$ that had not equilibrated [11]:

$$C_o\delta_o = \delta_{eq}(C_o - C_i) + f_{eq}C_i\delta_{eq} + (1 - f_{eq})C_i\delta_i \quad (2a)$$

$$f_{eq} = \frac{C_o\delta_o - C_i\delta_i - (C_o - C_i)\delta_{eq}}{C_i(\delta_{eq} - \delta_i)} \quad (2b)$$

In the absence of respiration C_o equals C_i and f_{eq} simplifies to $(\delta_o - \delta_i)/(\delta_{eq} - \delta_i)$, which would be the fraction without respiration correction. This respiration correction was small, with a 0.9% reduction in the median value of f_{eq}. The largest difference was 6% for the Carnegie Cornfield soils, which had the highest respiration rates. The abiotic sink for CO_2 in saline, alkaline desert soils outweighed rates of microbial respiration, causing an overestimation of f_{eq} with Eq. 2 of 1.9% in the median. Therefore, we did not apply the respiration correction for the desert soil samples from the Colorado River and Moab, Utah sites.

S4: Model framework for deriving CA activity in soils

Enzymatic (CA) activity from soil COS and $CO^{18}O$ exchange data was derived using a gas transport model that allowed to interpret the gas flux at the soil surface in terms of three processes occurring simultaneously within the soil matrix: production, diffusion and a first-order enzymatic reaction. In our model framework, and assuming steady state and homogeneous conditions, fair assumptions in our experimental conditions, the COS flux at the soil surface, F_{COS}, is expressed as [12]:

$$F_{COS} = \left(V_{d,COS} C_{COS} - z_{1,COS} P_{COS}\right) \tanh\left(\frac{z_{max}}{z_{1,COS}}\right), \quad (1)$$

where $V_{d,COS} = \sqrt{k_{h,COS} B_{COS} \theta D_{COS}}$, B_{COS} (m^3 m^{-3}) is the COS solubility in water [13], C_{COS} (mol m^{-3}) is the COS concentration above the soil surface, D_{COS} (m^2 s^{-1}) denotes the COS effective diffusivity through the soil matrix, P_{COS} (mol m^{-3} s^{-1}) is the COS production rate, $z_{1,COS} = D_{COS}/V_{d,COS}$ and z_{max} (m) is the maximum soil depth (i.e. soil thickness). $V_{d,COS}$ has the same dimension as a velocity (m s^{-1}) and can be seen as the maximum COS deposition velocity (i.e. in the absence of any production and for an infinite soil thickness), while $z_{1,COS}$ is referred to as the shallowest depth where COS hydrolysis dominates (above that depth diffusion out of the soil starts to compete with COS hydrolysis). The effective diffusivity D_{COS} was modeled according to the formulation of [14] for repacked soils as a function of volumetric soil moisture θ and porosity ϕ. Assuming that P_{COS} does not vary with soil moisture, F_{COS} will approach $P_{COS}z_{max}$ as soil moisture tends to zero. Taking COS flux data on dry soils as a proxy for $P_{COS}z_{max}$, we were then able to derive the CA-catalyzed rates for COS hydrolysis ($k_{h,COS}$, s^{-1}) from F_{COS} data on moist soils using the above equation and an iterative root finding method [12].

Using the same framework for CO_2 isotopes led to the same $CO^{18}O$ mass balance equation as in other studies [15, 16]. Assuming again steady state and homogeneous conditions, the $\delta^{18}O$ signature of the CO_2 flux at the soil surface, $\delta^{18}F$, could be derived [17]:

$$\delta^{18}O_F = \delta^{18}O_{eq} + \varepsilon_d\left(1 - \frac{z_{1,CO_2}}{z_{max}} \tanh\left(\frac{z_{max}}{z_{1,CO_2}}\right)\right) + \frac{V_{inv} C_{CO_2}}{F_{CO_2}}\left(\delta^{18}O_{eq} - \delta^{18}O_a\right) \tanh\left(\frac{z_{max}}{z_{1,CO_2}}\right), \quad (2)$$

where $V_{inv} = \sqrt{k_{iso} B_{CO_2} \theta D_{CO^{18}O}}$, B_{CO2} (m^3 m^{-3}) is the CO_2 solubility in water [18], C_{CO2} (mol m^{-3}) is the CO_2 concentration above the soil surface, $\delta^{18}O_a$ and $\delta^{18}O_{eq}$ (‰VPDB-CO_2) are the $^{18}O/^{16}O$ isotopic composition of CO_2 in the air above the soil surface and in equilibrium with soil water, D_{CO2} (m^2 s^{-1}) denotes the CO_2 effective diffusivity through the soil matrix and $z_{1,CO2} = D_{CO2}/V_{inv}$. V_{inv} has the dimension of a velocity (m s^{-1}) and is often referred to as the piston velocity while $z_{1,CO2}$ is referred to as the shallowest depth where full CO_2-H_2O equilibration takes place (above that depth diffusion out of the soil is too fast for full isotopic exchange to occur). As for COS, the effective diffusivity D_{CO2} was modeled according to the formulation of [14] for repacked soils. Using the above equation, we were then able to derive the CA-catalyzed rate for CO_2-H_2O isotopic exchange (k_{iso}, s^{-1}) from measurements of F_{CO2}, $\delta^{18}O_F$, $\delta^{18}O_a$ and $\delta^{18}O_{eq}$ on moist soils using an iterative root finding method[19]. Enhancement factors were calculated from f_{CA}= k_{cat} / $k_{uncat,std}$, where uncatalyzed rates at pH 4.5 and 20°C for CO_2 and COS are $k_{CO2,uncat,std}$=0.0057 s^{-1} [20] and $k_{COS,uncat,std}$=1.18e-5 s^{-1} [21], respectively.

S5: Extended molecular analyses

DNA and RNA extractions. Soil samples were collected from the moist soil incubations directly after gas exchange measurements for the extraction of DNA (flash-frozen in liquid nitrogen) and RNA (preserved in a 1:2 ratio of soil to LifeGuard® Soil Preservation Solution, MO BIO Laboratories, San Diego, CA, USA). Soil DNA was

extracted from 0.25 g of soil from each soil incubation replicate (20 sites, 3 replicates each) (PowerSoil® DNA Isolation Kit, MO BIO Laboratories, San Diego, CA, USA). We assessed DNA quantity fluorometrically (Qubit™ Fluorometer 2.0, Thermo Scientific, Waltham, MA, USA), purity using a full spectrum spectrophotometer (NanoDrop® ND-1000, Thermo Scientific, Waltham, MA, USA), and quality by gel electrophoresis. RNA was extracted from 1 g of soil in LifeGuard solution and extracted (PowerSoil® RNA Isolation Kit, MO BIO Laboratories, San Diego, CA, USA) from one replicate of the following samples: Big Basin, CA; Bondville, IL; Cambodian dry field; Carnegie corn field, CA; Jasper Ridge Sandstone; Great Plains, OK; Kohala Peninsula, HI; Stunt Ranch 1, CA; Stunt Ranch 2, CA; and Willow Creek, WI. RNA quantity and quality were assessed with the Agilent 2100 Bioanalyzer nano kit.

Sequencing and bioinformatics. Phylogenetic amplicon iTag DNA sequencing was performed using 16S rRNA (V4 region: 515F 5' GTGCCAGCMGCCGCGGTAA, 805R 5' GGACTACHVGGGTWTCTAAT-) and fungal ITS2 (ITS9F 5'GAACGCAGCRAAIIGYGA, ITS4R 5' TCCTCCGCTTATTGATATGC) primers (itagger analysis pool of 184, 2x250bp reads on Miseq). OTU tables (97% similarity for 16S rRNA and 95% for Fungal ITS2) were rarified to 40,000 and 80,000 sequences (GUniFrac R package [22](Chen, 2012) and community composition was visualized using non-metric multidimensional scaling (NMDS, metaMDS, Bray–Curtis dissimilarity [23]) and the principal coordinates plotted using the labdsv R package (pco [24]). Spearman correlations were used to identify statistically significant correlations between OTUs and environmental variables. A robust correlation was considered if the Spearman's correlation coefficient (ρ) was both $> |0.5|$ and statistically significant at AdjPValue $(q)<0.01$. Community diversity and richness metrics were calculated using alpha diversity estimates in Qiime v 1.9.1 and we report richness and diversity using the Observed OTUs and Shannon (H') index, respectively [25].

Metatranscriptome analysis. Metatranscriptomes were sequenced from soil RNA extract (2x150bp on HIseq 1T, 5 samples, 0.5 lanes per sample) at the Joint Genome Institute. Raw and assembled iTag and metatranscriptome data sets can be found on the JGI Genome Portal Website (https://genome.jgi.doe.gov/) under the JGI proposal ID 2033 study name "Microbial, chemical, and physical drivers of COS fluxes and ^{18}O-CO_2 exchange rates in soils".

References.

1. Fierer N, Schimel JP. Effects of drying-rewetting frequency on soil carbon and nitrogen transformations. *Soil Biol Biochem* 2002; **34**: 777–787.
2. Di Stefano C, Ferro V, Mirabile S. Comparison between grain-size analyses using laser diffraction and sedimentation methods. *Biosyst Eng* 2010; **106**: 205–215.
3. Beck T, Joergensen RG, Kandeler E, Makeshin E, Nuss E, Oberholzer HR, et al. An inter-laboratory comparison of ten different ways of measuring soil microbial biomass C. *Soil Biol Biochem* 1997; **29**: 1023–1032.
4. Brookes PC, Landman A, Pruden G, Jenkinson DS. Chloroform fumigation and the release of soil nitrogen: A rapid direct extraction method to measure microbial biomass nitrogen in soil. *Soil Biol Biochem* 1985; **17**: 837–842.
5. Harris I, Jones PD, Osborn TJ, Lister DH. Updated high-resolution grids of monthly climatic observations - the CRU TS3.10 Dataset. *Int J Climatol* 2014; **34**:

623–642.

6. Whelan ME, Hilton TW, Berry J a., Berkelhammer M, Desai AR, Campbell JE. Carbonyl sulfide exchange in soils for better estimates of ecosystem carbon uptake. *Atmos Chem Phys* 2016; **16**: 3711–3726.
7. Kooijmans LMJ, Uitslag NAM, Zahniser MS, Nelson DD, Montzka SA, Chen H. Continuous and high precision atmospheric concentration measurements of COS, CO_2, CO and H_2O using a quantum cascade laser spectrometer (QCLS). *Atmos Meas Tech Discuss* 2016; 1–36.
8. Commane R, Herndon SC, Zahniser MS, Lerner BM, McManus JB, Munger JW, et al. Carbonyl sulfide in the planetary boundary layer: Coastal and continental influences. *J Geophys Res Atmos* 2013; **118**: 8001–8009.
9. Bunk R, Behrendt T, Yi Z, Andreae MO, Kesselmeier J. Exchange of carbonyl sulfide (OCS) between soils and atmosphere under various CO 2 concentrations. *J Geophys Res Biogeosciences* 2017; **122**: 1343–1358.
10. Kaisermann A, Ogée J, Sauze J, Wohl S, Jones SP, Gutierrez A, et al. Disentangling the rates of carbonyl sulphide (COS) production and consumption and their dependency with soil properties across biomes and land use types. *Atmos Chem Phys Discuss* 2018; 1–27.
11. Von Sperber C, Weiler M, Brüggemann N. The effect of soil moisture, soil particle size, litter layer and carbonic anhydrase on the oxygen isotopic composition of soil-released CO 2. *Eur J Soil Sci* 2015; **66**: 566–576.
12. Ogée J, Sauze J, Kesselmeier J, Genty B, Van Diest H, Launois T, et al. A new mechanistic framework to predict OCS fluxes from soils. *Biogeosciences* 2016; **13**: 2221–2240.
13. Wilhelm E, Battino R, Wilcock RJ. Low-pressure solubility of gases in liquid water. *Chem Rev* 1977; **77**: 219–262.
14. Moldrup P, Yoshikawa S, Olesen T, Komatsu T, Rolston DE. Air Permeability in Undisturbed Volcanic Ash Soils. *Soil Sci Soc Am J* 2003; **67**: 32–40.
15. Tans PP. Oxygen isotopic equilibrium between carbon dioxide and water in soils. *Tellus, Ser B Chem Phys Meteorol* 1998; **50**: 163–178.
16. Wingate L, Ogée J, Burlett R, Bosc A, Devaux M, Grace J, et al. Photosynthetic carbon isotope discrimination and its relationship to the carbon isotope signals of stem, soil and ecosystem respiration. *New Phytol* 2010; **188**: 576–589.
17. Sauze J, Jones SP, Wingate L, Wohl S, Ogée J. The role of soil pH on soil carbonic anhydrase activity. *Biogeosciences Discuss* 2017; 1–28.
18. Weiss RF. Carbon dioxide in water and seawater: the solubility of a non-ideal gas. *Mar Chem* 1974; **2**: 203–215.
19. Press WH, Teukolsky SA, Vetterling WT, Flannery BP. Numerical Recipes in C: the Art of Scientific Computing. 1992. Cambrige University Press, Cambrige, UK.
20. Skirrow G. The dissolved gases-carbon dioxide. *Chem Oceanogr* 1975; **2**: 1–192.
21. Elliott S, Lu E, Rowland FS. Rates and mechanisms for the hydrolysis of carbonyl sulfide in natural waters. *Environ Sci Technol* 1989; **23**: 458–461.
22. Chen J. GUniFrac: Generalized UniFrac distances. R package version 1.0. 2012.
23. Oksanen J, Blanchet FG, Kindt R, Legendre P, Minchin PR, O'Hara RB, et al. vegan: Community Ecology Package. R package version 2.0-10. 2013.
24. Roberts DW. labdsv: Ordination and Multivariate Analysis for Ecology. R package

version 1.8-0. 2016.
25. Caporaso JG, Kuczynski J, Stombaugh J, Bittinger K, Bushman FD, Costello EK, et al. QIIME allows analysis of high-throughput community sequencing data. *Nat Methods* 2010; **7**: 335–6.
